# LIMK2 promotes the metastatic progression of triple-negative breast cancer by activating SRPK1

**DOI:** 10.1038/s41389-020-00263-1

**Published:** 2020-08-28

**Authors:** Parmanand Malvi, Radoslav Janostiak, Suresh Chava, Padmini Manrai, Esther Yoon, Kamaljeet Singh, Malini Harigopal, Romi Gupta, Narendra Wajapeyee

**Affiliations:** 1 Department of Biochemistry and Molecular Genetics and UAB O’Neal Comprehensive Cancer Center, The University of Alabama at Birmingham, Birmingham, AL 35294 USA; 2 Department of Pathology, Yale University School of Medicine, New Haven, CT 06510 USA; 3 Department of Pathology and Laboratory Medicine, Warren Alpert Medical School of Brown University, Providence, RI 02905 USA; 4 Present Address: Institute for Research in Biomedicine (IRB Barcelona), The Barcelona Institute of Science and Technology, 08028 Barcelona, Spain

**Keywords:** Breast cancer, Cancer genetics

## Abstract

Triple-negative breast cancer (TNBC) is a highly metastatic breast cancer subtype and due to the lack of hormone receptors and HER2 expression, TNBC has limited therapeutic options with chemotherapy being the primary choice for systemic therapy. LIM Domain Kinase 2 (LIMK2) is a serine/threonine kinase that plays an important role in the regulation of actin filament dynamics. Here, we show that LIM domain kinase 2 (LIMK2) is overexpressed in TNBC, and short-hairpin RNA (shRNA)-mediated LIMK2 knockdown or its pharmacological inhibition blocks metastatic attributes of TNBC cells. To determine the mechanism by which LIMK2 promotes TNBC metastatic progression, we performed stable isotope labeling by amino acids in cell culture (SILAC) based unbiased large-scale phosphoproteomics analysis. This analysis identified 258 proteins whose phosphorylation was significantly reduced due to LIMK2 inhibition. Among these proteins, we identified SRSF protein kinase 1 (SRPK1), which encodes for a serine/arginine protein kinase specific for the SR (serine/arginine-rich domain) family of splicing factors. We show that LIMK2 inhibition blocked SRPK1 phosphorylation and consequentially its activity. Furthermore, similar to LIMK2, genetic inhibition of SRPK1 by shRNAs or its pharmacological inhibition blocked the metastatic attributes of TNBC cells. Moreover, the pharmacological inhibition of LIMK2 blocked metastatic progression in mice without affecting primary tumor growth. In sum, these results identified LIMK2 as a facilitator of distal TNBC metastasis and a potential target for preventing TNBC metastatic progression.

## Introduction

Breast cancer is the most common cancer diagnosed in women, with an estimated 1.7 million new cases per year worldwide^1^. Molecular genetics studies have classified breast cancer into four major subtypes based on gene expression profiles: luminal A, luminal B, HER2-positive, and basal-like breast cancers^2^. Similar breast cancer subtypes were confirmed by The Cancer Genome Atlas studies^3^. Additionally, patient survival and response to therapies varies by breast cancer subtype^4^.

The majority of basal-like breast cancer tissue is characterized by the lack of hormone receptors and the absence of HER2 amplification; therefore, it is referred to as triple-negative breast cancer (TNBC)^5^. TNBC is an aggressive breast cancer accounting for 15–20% of all breast cancer cases^6^. TNBC is associated with a high mortality rate due in part to the highly metastatic nature of TNBC^7^, as well as the lack of effective therapies^8^. Therefore, understanding the mechanisms that drive TNBC progression and metastasis remains an important goal for effective management and treatment of this disease.

The LIM domain protein family consists of 65 human proteins, characterized by the presence of the LIM domain^9^. The LIM domain, a highly conserved cysteine-rich domain, participates in protein–protein interactions^10^. A smaller subfamily in this category includes the LIM domain kinase 2 (LIMK2), which is an important regulator of growth and invasion of several cancers, including pancreatic cancer, bladder cancer, osteosarcoma, and glioblastoma^11–15^. Additionally, some studies have shown that LIMK2 expression and activity increases after treatment with anticancer drugs, and these studies have implicated LIMK2 in drug resistance^16–18^.

Although LIMK2 has been implicated in several different cancer types, the role of LIMK2 in breast cancer is not fully understood. Furthermore, very few targets of LIMK2 have been identified. Of these, only cofilin 1 has been widely studied^19,20^. Here, we show that LIMK2 is overexpressed in TNBC and is necessary for the metastatic progression of TNBC. Using an unbiased global phosphoproteomics approach of stable isotope labeling with amino acids in cell culture (SILAC), we identified 258 proteins whose phosphorylation was significantly reduced due to LIMK2 inhibition, including SRSF protein kinase 1 (SPRK1). We found that LIMK2 inhibition blocks SRPK1 phosphorylation and activity; therefore, LIMK2 imparts a distal metastasis promoting effect, in part via regulating SRPK1 function.

## Results

### LIMK2 is overexpressed in TNBC and is necessary for facilitating TNBC metastatic attributes

**Fig. 1:**
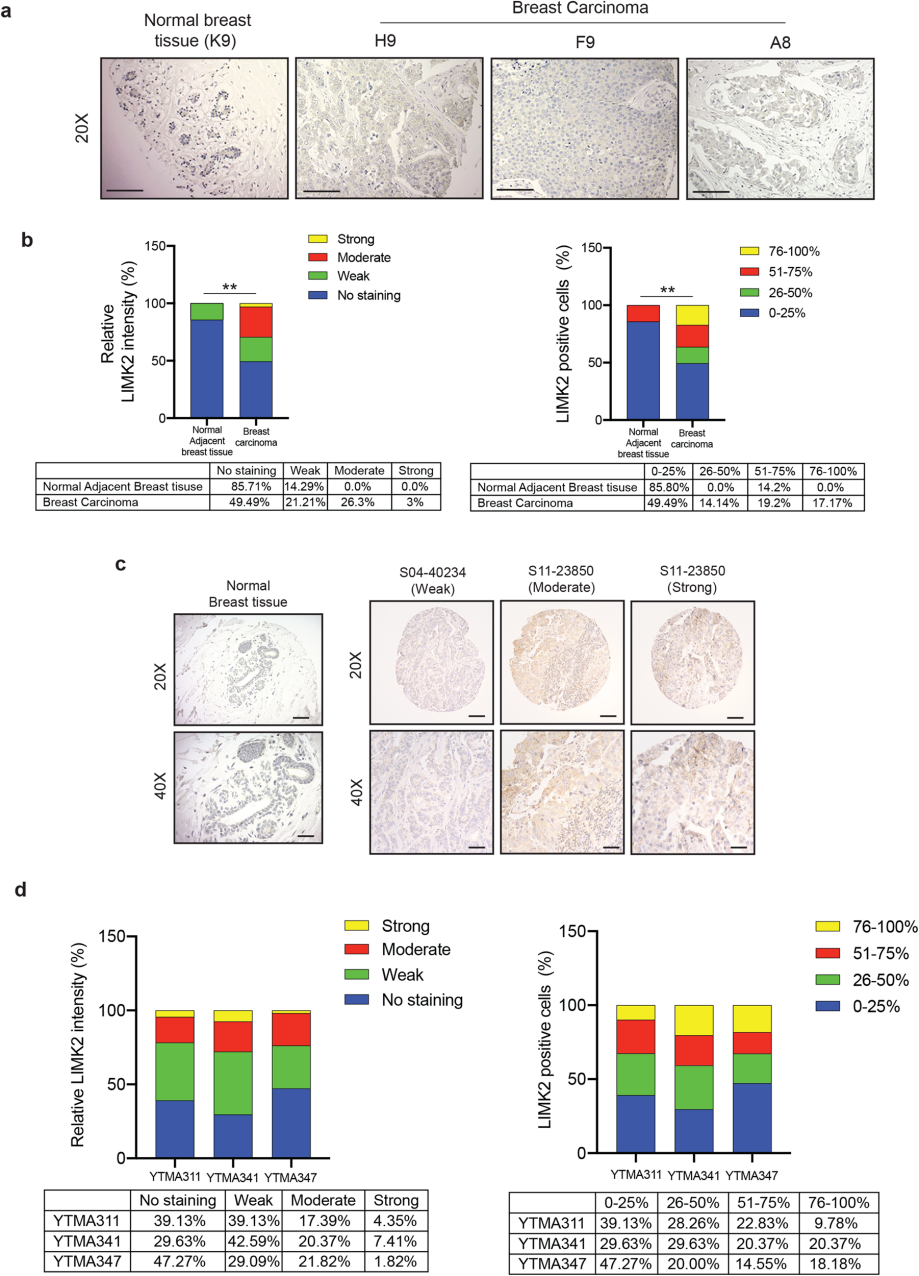
LIMK2 is overexpressed in triple-negative breast tumors. **a** Representative images for LIMK2 expression analysis in normal breast tissue or triple-negative breast cancer (TNBC) samples in a tissue microarray (TMA) from US Biomax at ×20 magnification. Scale bar, 50 μm. **b** (Left) Relative fraction of TNBC samples from US Biomax TMA scored by LIMK2 staining intensity: 0, no staining (not shown); +1, weak; +2, moderate; or +3, high. (Right) Percentage of LIMK2-positive cells in TNBC samples from US Biomax TMA: 0–25%, 25–50%, 51–75%, or 76–100%. Unpaired *t*-test with Welch’s correction was used to compare the LIMK2 expression between normal adjacent breast tissues and breast carcinoma. **c** Representative images for LIMK2 expression analysis in normal breast tissue or TNBC samples in three independent TNBC TMAs from Yale Tissue Microarray Facility (YTMA311, YTMA341, and YTMA347) at ×20 and ×40 magnification. Scale bar, 50 μm for ×20 and 25 μm for ×40. **d** (Left) Relative fraction of TNBC samples from three independent TNBC TMAs (YTMA311, YTMA341, and YTMA347) from Yale Tissue Microarray Facility scored by LIMK2 staining intensity: 0, no staining (not shown); +1, weak; +2, moderate; or +3, high. (Right) Percentage of LIMK2-positive cells in TNBC samples from three independent TNBC TMAs (YTMA311, YTMA341, and YTMA347) from Yale Tissue Microarray Facility: 0–25%, 25–50%, 51–75%, or 76–100%). **P < 0.01.

To understand the role of LIMK2 in breast cancer, we first asked whether LIMK2 is overexpressed in breast cancer. To this end, we analyzed a breast cancer tissue microarray (#BC081120e, US Biomax Inc.). This tissue array included 100 cases of invasive breast cancer and 10 adjacent normal breast tissues with information on ER, PR, and HER2 status (Table S1). In immunohistochemical analysis, we found that LIMK2 expression was significantly higher in breast cancer samples, including those of TNBC, than in normal tissue (Fig. S1a, b, Table S1, Figs. 1a, b, and S1c). Because TNBC is an aggressive breast cancer subtype that currently lacks effective therapeutic approaches, we focused our studies with LIMK2 on TNBC. To further establish the role of LIMK2 in TNBCs, we expanded our LIMK2 protein expression analyses. To this end, we analyzed three additional TNBC tissue microarrays for LIMK2 protein expression using immunohistochemistry (IHC): YTMA311 (*n* = 92), YTMA341 (*n* = 53), and YTMA347 (*n* = 54; Table S2). Similar to the results of our initial analysis, we found that a subset of TNBC patient samples also expressed higher levels of LIMK2 (Fig. 1c, d and Table S2). Consistent with our results, analysis of multiple publicly available breast cancer mRNA expression datasets also showed significantly higher LIMK2 mRNA expression in TNBC (ERBB2/ER/PR negative) compared to breast cancer with other biomarker status (Fig. S1d–i)^21–26^. Furthermore, by analyzing several publicly available breast cancer mRNA expression datasets, we found that increased LIMK2 expression was also associated with increased incidence of metastasis, recurrence, and death in breast cancer patients (Fig. S2).

**Fig. 2:**
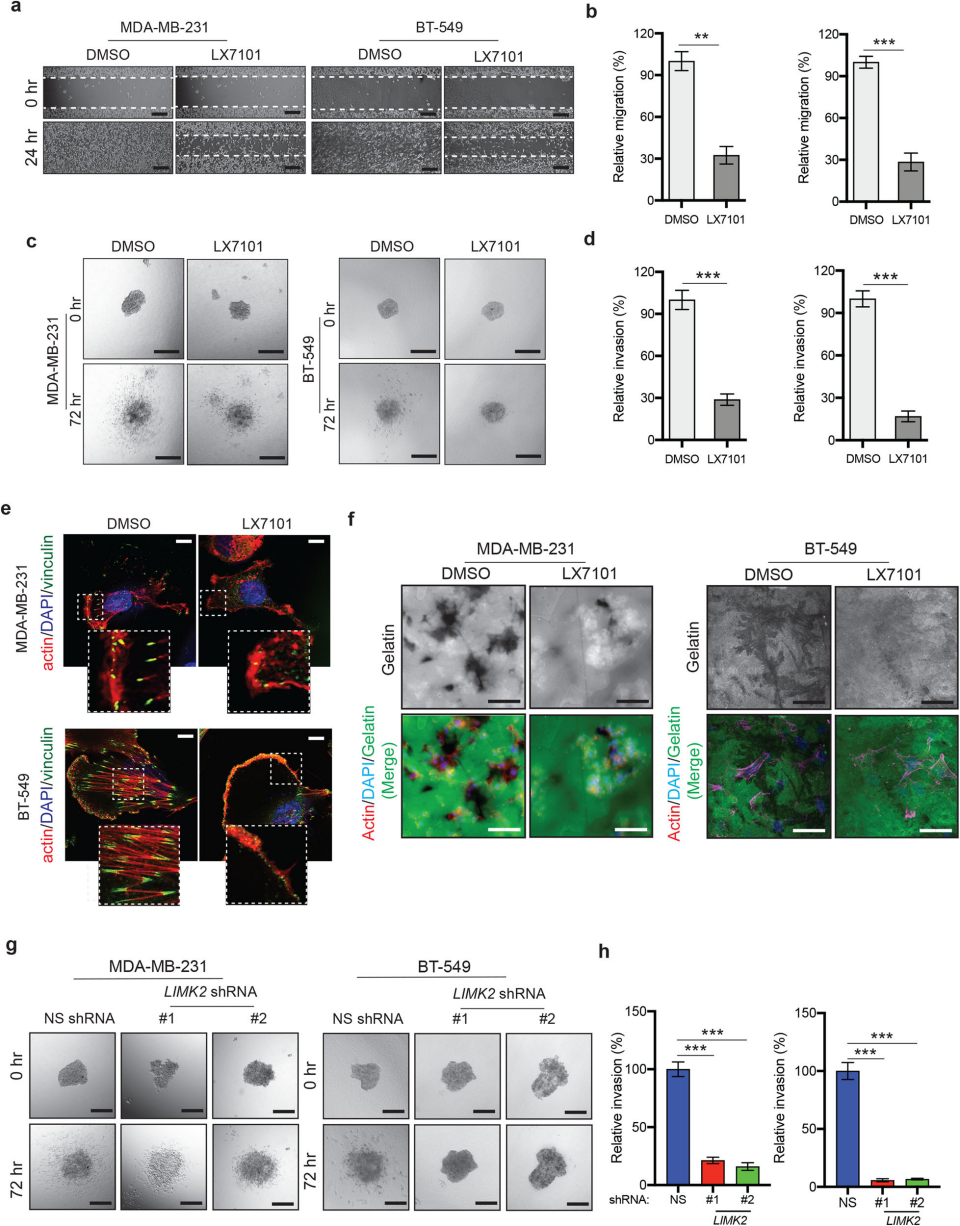
LIMK2 facilitates metastatic attributes of triple-negative breast cancer (TNBC) cells. **a** MDA-MB-231 and BT-549 cells treated with the vehicle or LX7101 (5 μM) were analyzed using a wound healing assay. Representative images at the indicated times are shown. Scale bar, 250 µm. **b** Relative migration (%) calculated from the data presented in **a**. **c** MDA-MB-231 and BT-549 cells treated with the vehicle or LX7101 (5 μM) were analyzed using a 3D spheroid invasion assay. Representative images at the indicated times are shown. Scale bar, 250 µm. **d** Relative invasion (%) calculated from the data presented in **c**. **e** MDA-MB-231 and BT-549 cells were stained with phalloidin (red), vinculin (green), and DAPI (blue) after treatment with the vehicle or LX7101. Scale bar, 5 µm. **f** Extracellular matrix degradation capacity of MDA-MB-231 and BT-549 cells treated with the vehicle or LX7101 (5 μM) was analyzed using the gelatin degradation assay. Cells were stained with phalloidin (red) and DAPI (blue). Gelatin (green) degradation appears as black areas. Representative images are shown. Scale bar, 500 µm. **g** 3D spheroid invasion assay in MDA-MB-231 and BT-549 cells expressing *LIMK2* shRNA. Representative images at the indicated times are shown. Scale bar, 250 µm. **h**. Relative invasion (%) calculated from the data presented in **g**. Data are represented as the means ± SD. **P < 0.01; ***P < 0.001.

Next, based on the results of our IHC and guided by the previous work on the role of LIMK2 in metastasis^12,18,27–29^, we asked whether inhibition of LIMK2 attenuates the metastatic characteristics of TNBC cells. To test this, we used a small-molecule LIMK2 inhibitor, LX7101 (Lexicon Pharmaceutical), to determine whether pharmacological inhibition of LIMK2 altered the metastatic properties of breast cancer cells. LX7101 has also shown in vivo efficacy for treating glaucoma and is currently being tested in phase 1 clinical trials^30^. To measure the effect of LX7101 on the metastatic attributes of TNBC cells, we treated TNBC cell lines MDA-MB-231 and BT-549 with LX7101 and assessed migration, invasiveness, actomyosin contractility, and extracellular matrix (ECM) degradation using three independent assays. As expected, LX7101 treatment resulted in reduced phosphorylation of the LIMK2 substrate cofilin (pSer3; Fig. S3a), reduced migration (Fig. 2a, b), reduced invasion (Fig. 2c, d), increased disassembly of focal adhesions and associated stress fibers (Fig. 2e), and reduced gelatin degradation by TNBC cells compared with dimethyl sulfoxide (DMSO)-treated TNBC cells (Fig. 2f).

To further strengthen the results obtained using LX7101, we used another LIMK2 small-molecule inhibitor, TH-257, a type III allosteric LIMK2 inhibitor that targets LIMK2 DGF-out conformation in a non-ATP-competitive manner and that does not exhibit significant off-target activity by KINOMEscan assay at 1 μM^31^. TH-257 also significantly inhibited the metastatic characteristics of TNBC cells in various cell-based assays (Fig. S3b–g).

Small-molecule inhibitors can inhibit more than one target, albeit at different specificities; therefore, to further confirm the results obtained using small-molecule inhibitors of LIMK2 (LX7101 and TH-257), we knocked down the expression of *LIMK2* in TNBC cells (MDA-MB-231 and BT-549) using short-hairpin RNAs and analyzed the effect of the *LIMK2* genetic knockdown on their ability to invade in a 3D invasion assay. In agreement with our results with LIMK2 inhibitors (LX7101 and TH-257), the genetic knockdown of *LIMK2* using shRNAs also resulted in significantly reduced invasion of TNBC cells (Figs. 2g, h and S3h, i). Collectively, these results demonstrate that LIMK2 is overexpressed in TNBC cells and is necessary for maintaining metastatic attributes in TNBC cells.

### SILAC identifies proteins with reduced phosphorylation following LIMK2 inhibition

**Fig. 3:**
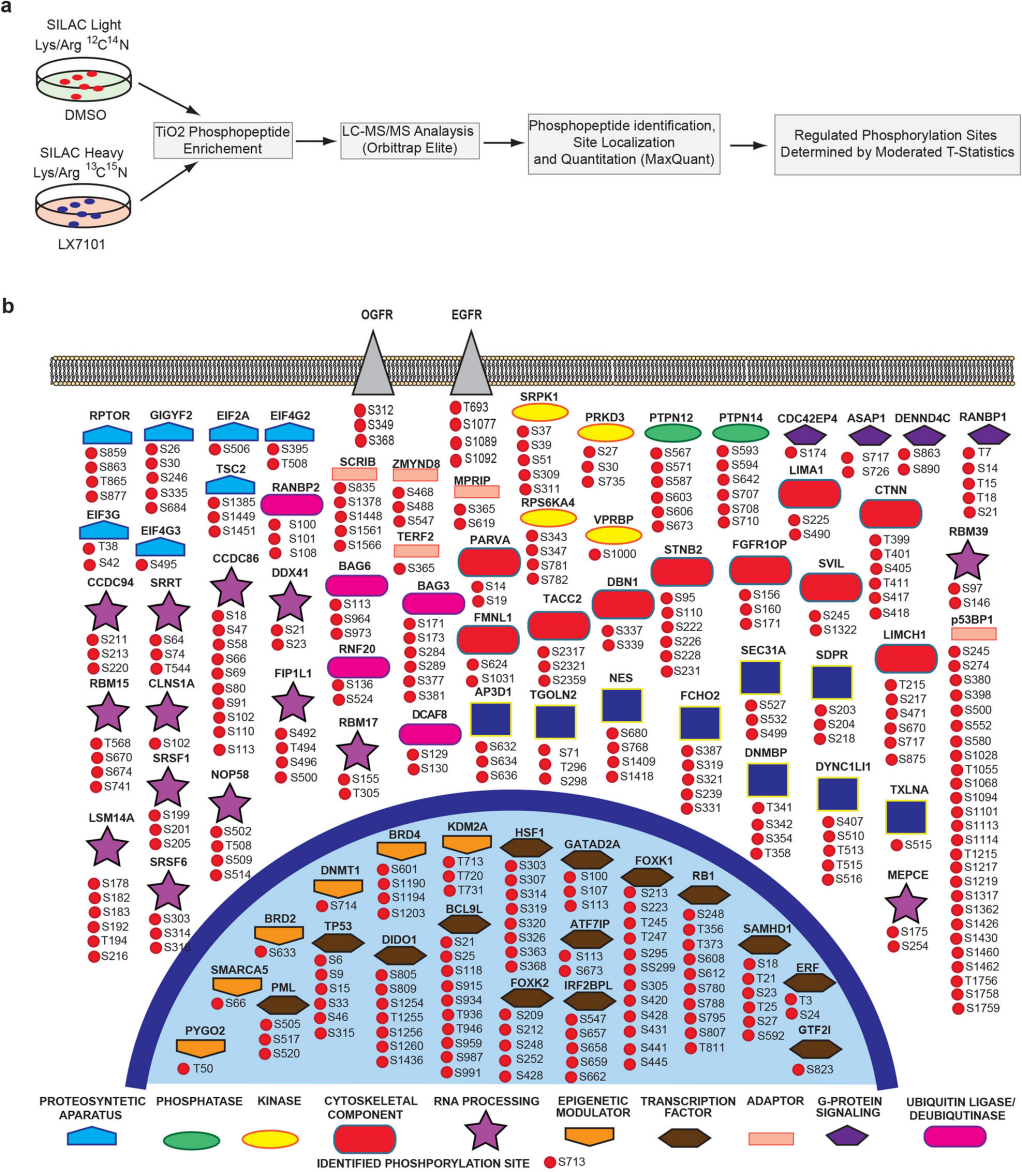
SILAC identifies proteins whose phosphorylation is reduced due to LIMK2 inhibition. **a** Schematic representation of the major steps of SILAC analysis to identify phosphopeptides that are altered after treatment with LX7101 in MDA-MB-231 cells. **b** Functional classification, localization, and phosphorylation sites for a subset of proteins that show reduced phosphorylation after LX7101 treatment.

Because LIMK2 is a serine/threonine kinase, we hypothesized that LIMK2 exerts its metastasis-promoting activities by regulating the phosphorylation of its downstream targets. However, downstream mediators of LIMK2 are largely unknown, and few downstream substrates have been identified^19,20,32^. Therefore, to comprehensively identify proteins whose phosphorylation is reduced by LIMK2 inhibition, we took the unbiased phosphoproteomics approach of SILAC^33^. For SILAC analysis, MDA-MB-231 cells were cultured in either light culture medium containing lysine and arginine labeled with light carbon (^12^C) and light nitrogen (^14^N), or cultured in heavy medium containing lysine and arginine labeled with heavy carbon (^13^C) and heavy nitrogen (^15^N). After five cell doublings, MDA-MB-231 cells in light medium were treated with DMSO, and cells in heavy medium were treated with LX7101 for 6 h (Fig. 3a). SILAC identified 258 proteins whose phosphorylation was significantly reduced due to LIMK2 inhibition (Table S3). These proteins belong to several different biological pathways (Fig. 3b).

**Fig. 4:**
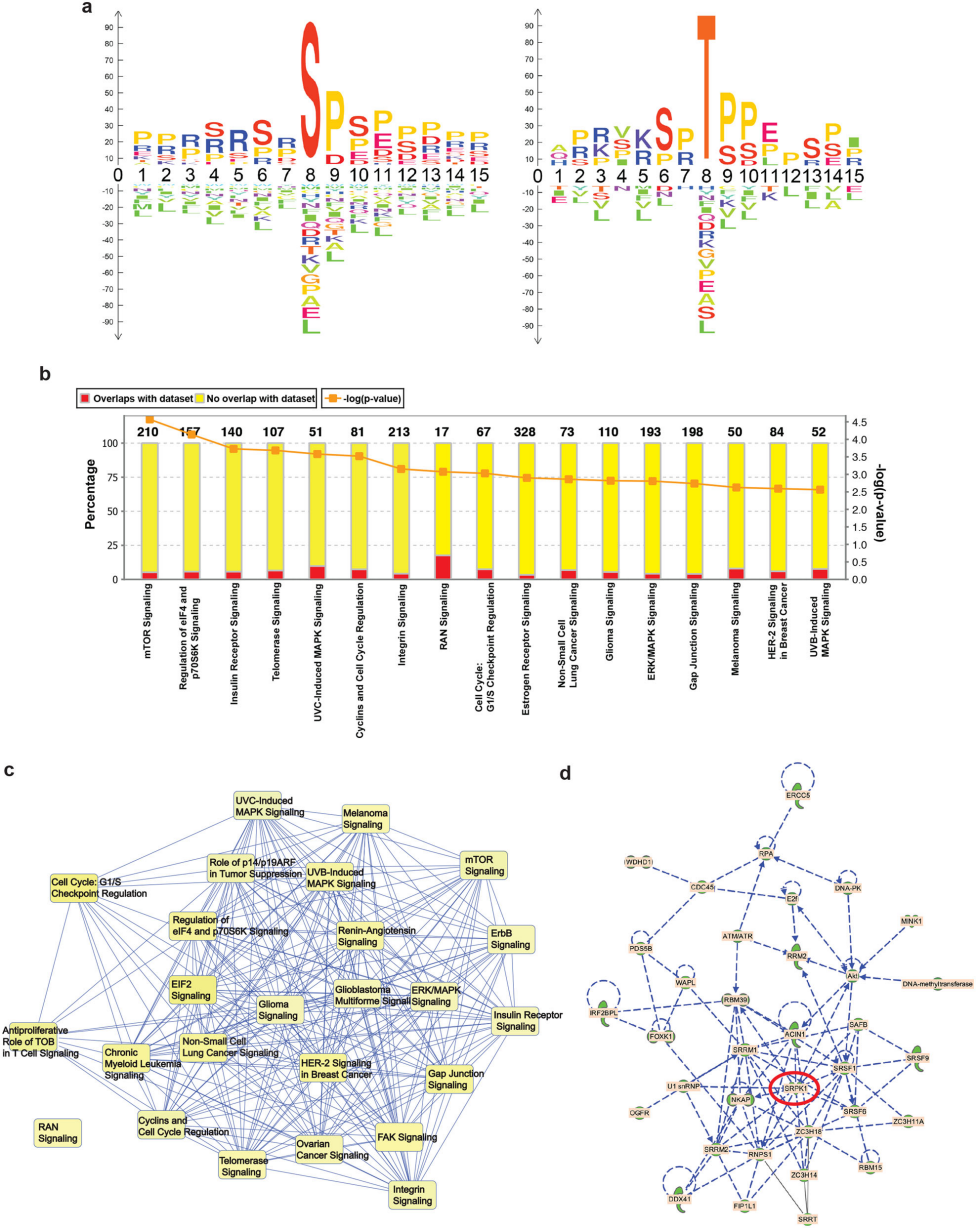
Ingenuity pathway analysis reveals key pathways altered due to LIMK2 inhibition. **a** LIMK2 phosphorylation site consensus sequence identified by dagLogo package. **b** Top 10 signaling pathways identified using IPA analysis on SILAC data. **c** Overlap of signaling network regulated by LIMK2 identified by IPA software. **d** Schematic diagram illustrating the SRPK1-regulated signaling network.

We next analyzed the SILAC data to predict the preferred amino acid motif for LIMK2-induced phosphorylation using a newly developed method (R/Bioconductor package dagLogo). Using this approach, we identified serine or threonine followed by 2 prolines or serine as the LIMK2 preferred phosphorylation motif in our SILAC studies (Fig. 4a). We also analyzed the SILAC data using ingenuity pathway analysis (IPA) to functionally classify the proteins with reduced phosphorylation after LIMK2 inhibition. IPA analysis revealed proteins in several pathways including ones that regulate integrin signaling (Fig. 4b–d and Tables S4 and S5), a result that is consistent with the important role of LIMK2 for stimulating metastatic attributes. In particular, we observed reduced phosphorylation of SRPK1 (Fig. 4d). SRPK1 promotes metastatic attributes in several cancer types, including breast cancer^32,34–37^. Thus, we focused our studies on investigating the role of SRPK1 in regulating the ability of LIMK2 to facilitate the metastatic attributes of TNBC cells.

### SRPK1 is overexpressed in TNBC and its inhibition impairs metastatic attributes of TNBC cells

**Fig. 5:**
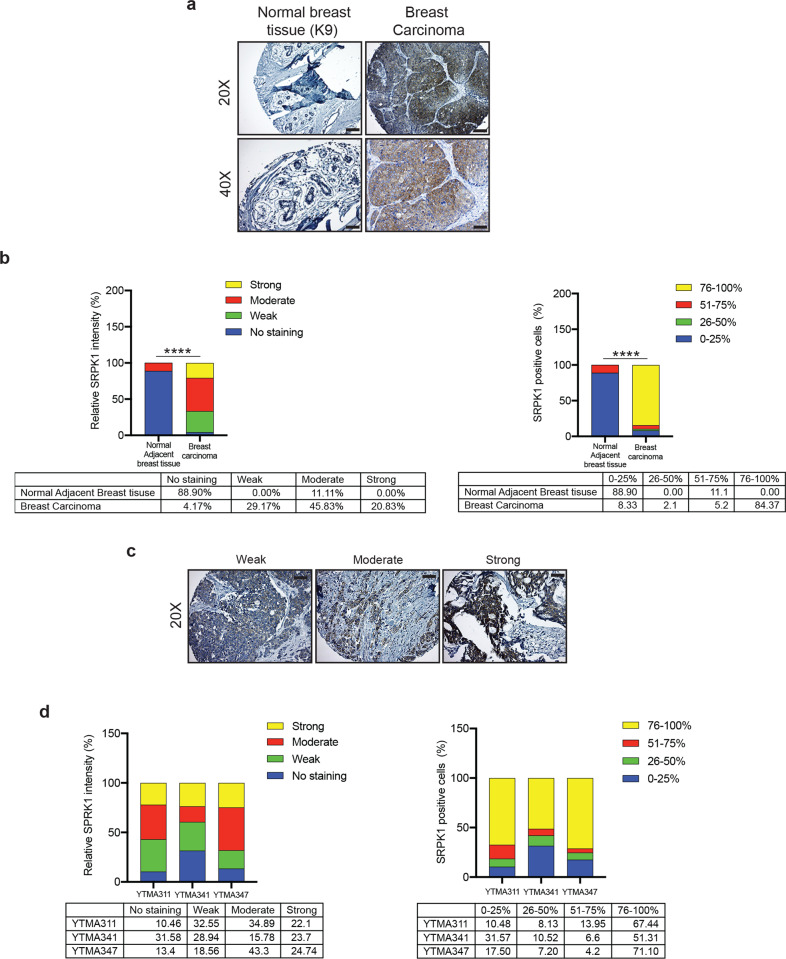
SRPK1 is overexpressed in triple-negative breast cancer tumors. **a** Representative images for SRPK1 expression analysis in normal breast tissue and triple-negative breast cancer (TNBC) samples on a tissue microarray (TMA) from US Biomax at 20× and 40× magnification. Scale bar, 50 μm for ×20 and 25 μm for ×40. **b** (Left) Relative fraction of TNBC samples from US Biomax TMA scored based on SRPK1 staining intensity: 0, no staining (not shown); +1, weak; +2, moderate; or +3, high. (Right) percentage of SRPK1-positive cells in TNBC samples from US Biomax TMA: 0–25%, 25–50%, 51–75%, or 76–100%. Unpaired *t*-test with Welch’s correction was used to compare the SRPK1 expression between normal adjacent breast tissues and breast carcinoma. **c** Representative images for SRPK1 expression analysis in normal breast tissue or TNBC samples from three independent TNBC TMAs from the Yale Tissue Microarray Facility (YTMA311, YTMA341, and YTMA347) at ×20 magnification. Scale bar, 50 μm. **d** (Left) Relative fraction of TNBC samples from three independent TNBC TMAs (YTMA311, YTMA341, and YTMA347) from the Yale Tissue Microarray Facility scored by SRPK1 staining intensity: 0, no staining (not shown); +1, weak; +2, moderate; or +3, high. (Right) percentage of SRPK1-positive cells in TNBC samples from three independent TNBC TMAs (YTMA311, YTMA341, and YTMA347) from the Yale Tissue Microarray Facility: 0–25%, 25–50%, 51–75%, or 76–100%. ****P < 0.0001.

SRPK1 was one of the proteins identified from SILAC analysis displaying reduced phosphorylation following LX7101 treatment. SRPK1 has been previously implicated in the regulation of breast cancer metastasis^34^. To define the role of SRPK1 in breast cancer, we first asked whether SRPK1 is overexpressed in breast cancer. To this end, we assessed SRPK1 expression using the US BioMax tissue microarray (#BC081120e) and found that SRPK1 expression was significantly higher in breast cancer samples, including TNBC samples, compared to normal adjacent tissue (Table S6 and Fig. 5a, b). To further establish the role of SRPK1 in TNBCs, we analyzed three additional TNBC tissue microarrays for SRPK1 protein expression using IHC: YTMA311 (*n* = 92), YTMA341 (*n* = 53), and YTMA347 (*n* = 54; Table S7). In support of our initial SRPK1 expression analysis, we found that TNBC patient samples expressed higher levels of SRPK1 protein than did normal breast tissue (Fig. 5c, d and Table S7). Consistent with our results, analysis of multiple, publicly available breast cancer mRNA expression datasets also showed significantly higher SRPK1 mRNA expression in TNBC (ERBB2/ER/PR negative) samples compared to non-TNBC breast cancers (Fig. S4). Furthermore, by analyzing several publicly available datasets of breast cancer mRNA expression datasets, we found that increased SRPK1 expression correlated with an increased incidence of metastasis, recurrence, and death in breast cancer patients (Fig. S5). Based on these collective findings, we decided to focus on SRPK1 for further studies.

**Fig. 6:**
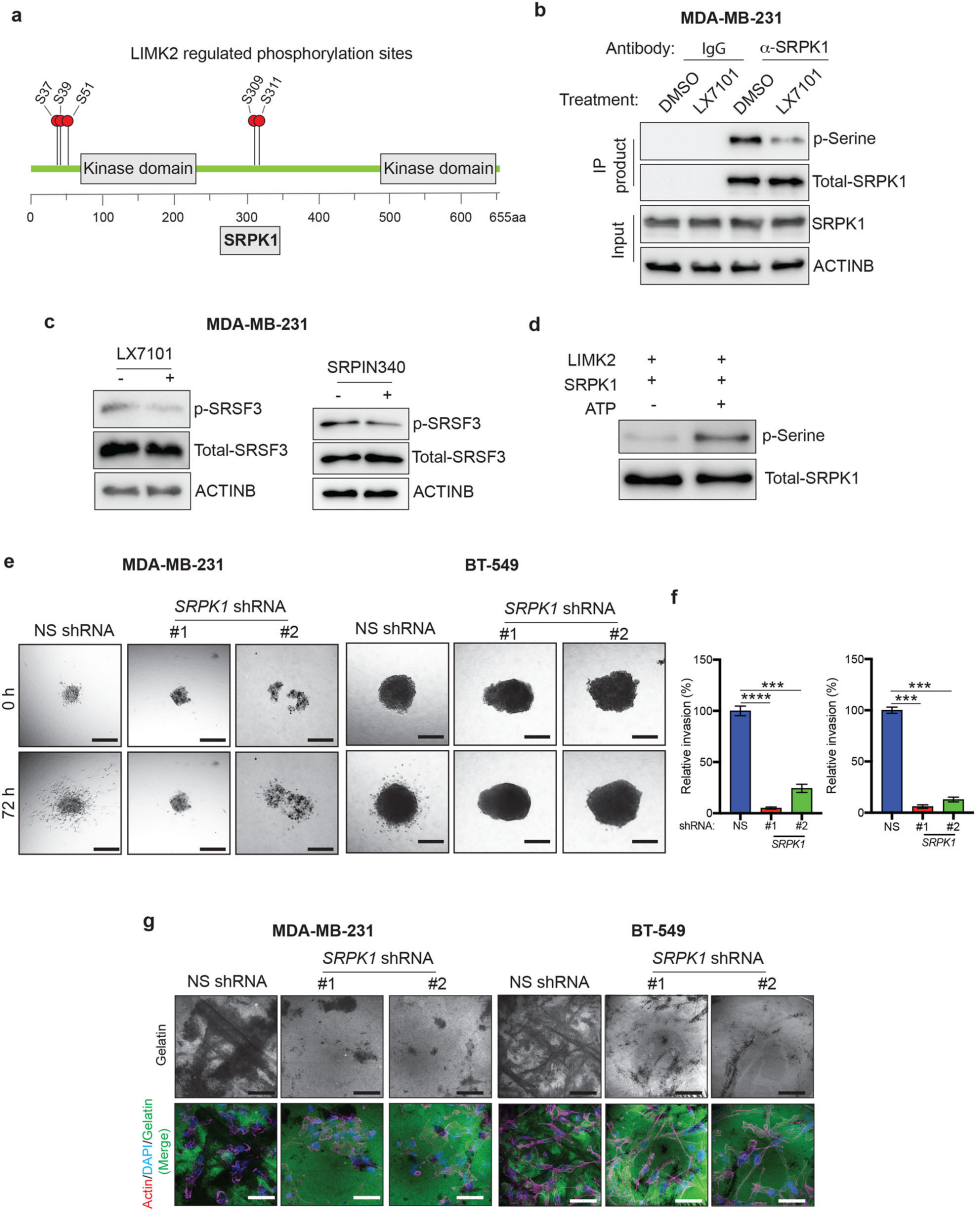
SRPK1 is an important downstream mediator of LIMK2. **a** Schematic representation of SRPK1 protein with its identified phosphorylation sites. **b** MDA-MB-231 cells were treated with either LX7101 (5 μM) or vehicle control for 6 h. SRPK1 was immunoprecipitated from LX7101- and vehicle-treated-MDA-MB-231 cells. Immunoprecipitates were analyzed by immunoblotting with pan-phospho serine antibodies and beta-actin antibodies (loading control). **c** MDA-MB-231 cells were treated with LX7101 (5 μM), SRPK1 inhibitor SRPIN340 (10 μM), or vehicle control for 6 h. Expression of the indicated proteins was measured by immunoblotting. Beta-actin was used as a loading control. **d** LIMK2 in vitro kinase assays were performed in the presence and absence of ATP (1 mM). The samples were analyzed by immunoblotting for serine phosphorylation and total SRPK1. **e** TNBC cell lines (MDA-MB-231 and BT-549) expressing *SRPK1* shRNA were analyzed using a 3D spheroid invasion assay. Representative images taken at the indicated times are shown. Scale bar, 250 µm. **f** Relative invasion (%) calculated from the data presented in **e**. **g** Extracellular matrix degradation capacity of TNBC cell lines (MDA-MB-231 and BT-549) expressing *SRPK1* shRNA was analyzed using a gelatin degradation assay. Cells were stained with phalloidin (red) and DAPI (blue). Gelatin (green) degradation appears as black areas. Representative images are shown. Scale bar, 500 µm. Data are represented as the means ± SD. ***P < 0.001; ****P < 0.0001.

SILAC analysis revealed five phosphorylation sites on SRPK1 (Ser7, Ser9, Ser51, Ser309, and Ser311) that showed reduced phosphorylation after LX7101 treatment (Fig. 6a). To confirm that LIMK2 inhibition also reduced SRPK1 phosphorylation in TNBC cells, we used immunoprecipitation experiments. MDA-MB-231 cells were treated with LX7101 and the proteins were immunoprecipitated with anti-SRPK1 antibodies. The phosphorylation status of immunoprecipitated SRPK1 was assessed by immunoblotting with a pan-specific anti-phosphoserine antibody. We found that treatment of MDA-MB-231 cells with LX7101 results in reduced serine phosphorylation of SRPK1 (Fig. 6b). To extend this analysis, we tested whether inhibition of LIMK2 or SRPK1 leads to reduced phosphorylation of downstream targets of SRPK1. We observed that inhibition of LIMK2 by LX7101 or of SRPK1 by a small-molecule inhibitor of SRPK1, SRPIN340, reduced the phosphorylation of the SRPK1 target protein Serine and Arginine Rich Splicing Factor 3 (SRSF3; Fig. 6c). Finally, we asked whether LIMK2 might directly phosphorylate SRPK1. In vitro kinase assays revealed that LIMK2 phosphorylates SRPK1 (Fig. 6d). These results suggest that the activity of SRPK1 is regulated by LIMK2; therefore, SRPK1 might function downstream of LIMK2 to affect the metastatic attributes of TNCB cells.

To determine whether *SRPK1* knockdown mimics the phenotypes associated with LIMK2 inhibition, we knocked down the expression of SRPK1 in TNBC cell lines (MDA-MB-231, BT-549, MDA-MB-468; Fig. S6a, b). SRPK1 knockdown cells were then analyzed using 3D invasion and ECM degradation assays. Our results showed that *SRPK1* knockdown impaired invasion in 3D collagen (Figs. 6e, f and S6c, d) and led to decreased degradation of ECM (Figs. 6g and S6e) in multiple TNBC cell lines.

**Fig. 7:**
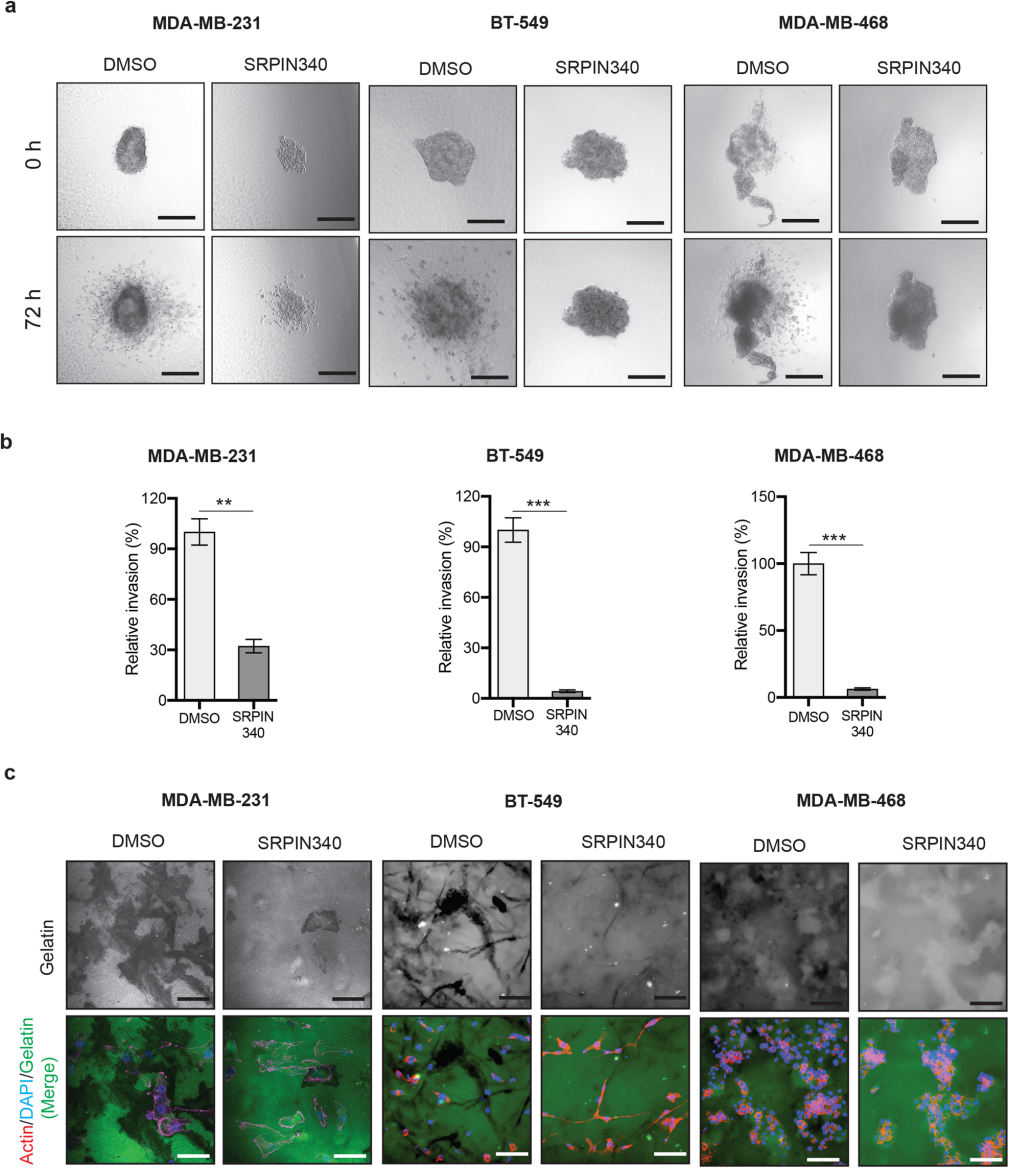
SRPK1 is important for metastatic attributes of TNBC cells. **a** 3D spheroid invasion assay in TNBC cell lines (MDA-MB-231, BT-549, and MDA-MB-468) treated with SRPK1 inhibitor SRPIN340 (10 µM) or vehicle control. Representative images taken at the indicated times are shown. Scale bar, 250 µm. **b** Relative invasion (%) calculated from the data presented in **a**. **c** Extracellular matrix degradation capacity of TNBC cell lines (MDA-MB-231, BT-549, and MDA-MB-468) treated with SRPIN340 (10 µM) or vehicle control was analyzed using the gelatin degradation assay. Cells were stained with phalloidin (red) and DAPI (blue). Gelatin (green) degradation appears as a black area. Representative images are shown. Scale bar, 500 µm. Data are represented as the means ± SD. **P < 0.01; ***P < 0.001.

Finally, to corroborate the results obtained using *SRPK1* shRNAs, we used a small-molecule inhibitor of SRPK1, SRPIN340, on 3D invasion and ECM degradation in TNBC cells (MDA-MB-231, BT-549, and MDA-MB-468). SRPIN340 is an ATP-competitive serine-arginine-rich protein kinase inhibitor. Consistent with the results we obtained with *SRPK1* genetic knockdowns using shRNAs, the pharmacological inhibition of SRPK1 by SRPIN340 also resulted in reduced invasion (Fig. 7a, b) and led to decreased degradation of ECM (Fig. 7c) in multiple TNBC cell lines. Collectively, these results demonstrate that SPRK1 inhibition resulted in phenotypes similar to those observed with LIMK2 inhibition, and that SPRK1 inhibition reduced the metastatic properties of TNBC cells.

### Pharmacological inhibition of LIMK2 inhibits metastatic progression in mice

**Fig. 8:**
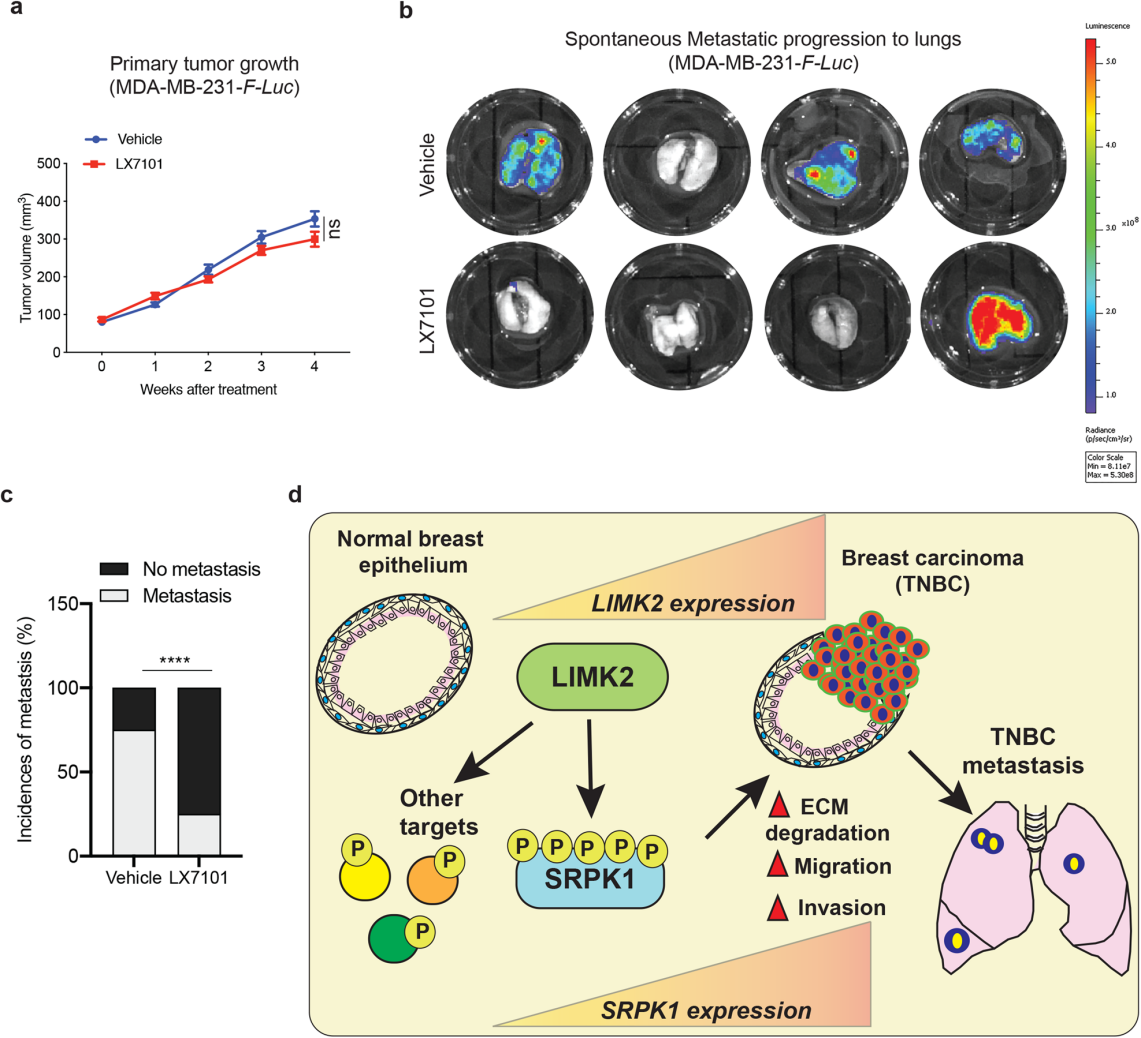
Pharmacological inhibition of SPRK1 or LIMK2 inhibits metastatic characteristics of TNBC. **a** Firefly luciferase–labeled MDA-MB-231 cells (MDA-MB-231-*F-Luc*) were injected subcutaneously into NSG mice (*n* = 4/group). From week 4, mice were treated orally with the vehicle (0.5% methyl cellulose in PBS) or 50 mg/kg LX7101 (in 0.5% methyl cellulose in PBS) every other day. Tumor volumes for mice were measured and plotted each week. Week 4 data includes four vehicle-treated mice and four drug-treated mice. **b** Imaged lungs of the mice killed at the end of the experiment presented in **a**. **c** Percentage incidence of metastasis is presented in indicated groups. Contingency analysis using Fisher’s exact test shows statistically significant difference in the incidence of metastasis between vehicle or LX7101 treatment. **d** A model showing the mechanism by which LIMK2 facilitates metastatic progression of TNBC. The model indicates that LIMK2 is necessary for distal metastasis of TNBC and functions, in part, by phosphorylating and increased the kinase activity of SRPK1. Data in **a** is presented as the means ± SD. ns not significant *P*-value, ****P < 0.0001.

Because we found that both genetic and pharmacological inhibition of LIMK2 blocked metastatic properties in cell culture-based assays, we asked whether LIMK2 inhibition using LX7101 could block the metastatic progression of TNBC cells in mice. To this end, we injected mice subcutaneously with MDA-MB-231 cells labeled with firefly luciferase. This was followed by treatment with either vehicle or the LIMK2 inhibitor LX7101. Throughout the experiment, tumor growth was measured each week. At the end of the experimental period, the animals were killed and the lungs were dissected and analyzed with bioluminescence imaging to measure spontaneous metastatic progression to the lungs. We found that LX7101 treatment did not inhibit tumor growth (Fig. 8a). However, mice treated with LX7101 showed spontaneous metastasis in only 1 out of 4 mice (25%), whereas vehicle-treated mice showed spontaneous metastasis in 3 out of 4 mice (75%; Fig. 8b, c). These results demonstrate the in vivo role of LIMK2 in distal metastatic progression and indicate that pharmacological inhibition of LIMK2 might be a useful therapeutic approach for preventing distal metastatic progression of TNBC.

## Discussion

Breast cancer remains the leading cause of cancer-related death in women. Among the breast cancer subtypes, TNBC remains a significant clinical challenge due to the relative ineffectiveness of current chemotherapeutic options and a paucity of actionable drug targets. In this study, we showed that LIMK2 kinase is overexpressed in TNBC and that both genetic and pharmacological inhibition of LIMK2 blocks TNBC metastatic progression due, in part, to its ability to phosphorylate SRPK1 and possibly other downstream targets (Fig. 8d).

We found that LIMK2 was overexpressed in various breast cancer subtypes, including a substantial portion of TNBCs, using tissue microarrays. We corroborated this finding using publicly available datasets to show that LIMK2 expression was higher in TNBC tumors compared to other breast cancer subtypes and to show that LIMK2 overexpression predicted increased incidence of metastasis, disease recurrence, and death in breast cancer patients. These findings are consistent with those of a previous study that showed that elevated LIMK2 expression correlated with larger tumor size and higher histological grade and was a predictor of worse disease-free survival (DFS) and overall survival (OS)^38^. Multivariable Cox regression analysis indicated elevated expression of LIMK2 was an independent prognostic factor for both DFS and OS^38^.

LIMK2 was previously implicated in promoting metastatic behavior in various cancer models. For example, a previous study showed knockdown of *LIMK2* reduced pancreatic cancer cell-induced angiogenesis^14^. Another study showed that the selective LIMK2 inhibitor T56-LIMKi blocked pancreatic cancer xenograft growth in mice^39^. Additionally, a colorectal cancer study found that the LIMK/cofilin pathway is associated with colorectal cancer progression and chemoresistance^18^. Additionally, imbalance of LIMK1 and LIMK2 expression leads to human colorectal cancer progression and metastasis via promoting the nuclear translocation of β-catenin^11^. We found that LIMK2 was required for maintaining the metastatic characteristics of TNBC cells because both genetic and pharmacological inhibition of LIMK2 inhibited their metastatic attributes. The finding that LIMK2 influences metastatic attributes but not tumor growth is intriguing, but not unprecedented. Other studies have also identified genes that primarily promote metastasis without affecting primary tumor growth^40,41^, although the underlying mechanisms were not clear. Therefore, why some genes promote only tumor growth or metastasis remains an open question.

Based on our results, we predict that LIMK2 promotes metastasis, but not tumor growth because the primary effect of LIMK2 is on phenotypes related to metastasis, such as invasion and migration. These characteristics are not required for tumor growth but are essential for metastatic progression. This idea is further supported by our mouse experiments, in which the treatment of MDA-MD-231 xenografts with the LIMK2 inhibitor LX7101 did not suppress primary tumor growth but did inhibit metastatic progression. Collectively, these results show that LIMK2 facilitates metastasis in TNBC.

LIMK2 is a serine/threonine protein kinase for which only a limited number of downstream meditators have been identified^19,20,32^. Prior studies have undertaken targeted approaches, rather than more comprehensive, unbiased approaches, like SILAC, to identify LIMK2 targets. Targeted approaches identified cofilin as a substrate of LIMK2. LIMK2 impairs the actin-severing activity of Cofilin by phosphorylating it at Serine 3, which causes actin stabilization^20,42–44^. Therefore, LIMK2 inhibition hyperactivates cofilin and increases actin turnover, leading to the disruption of actin stress fibers and their associated focal adhesions^20^. Furthermore, LIMK2 phosphorylates the MT1-MMP protease on tyrosine 573, which enhances its association with cortactin, resulting in increased degradation of extracellular matrix and stimulation of invasion^28^.

In contrast to these prior studies, we applied the unbiased phosphoproteomics approach of SILAC to identify proteins with reduced phosphorylation following LIMK2 inhibition. We identified 258 proteins displaying significantly reduced phosphorylation levels. As expected, based on the known functions of LIMK2, many of the affected proteins were in pathways that regulate the actin cytoskeleton, integrin signaling, or cell polarity (PARVA, RRAS2, MPRIP, PAK2, ILKAP, and SCRIB). In addition, several functionally diverse protein groups, such as kinases (SRPK1 and RPS6KA4), phosphatases (PTPN12 and PTPN14), transcription factors (HSF1 and p53), or splicing factors (SRSF1 and SRSF6) were also identified. Furthermore, LIMK2 inhibition also affected mRNA processing (SRPK1, SRRM1/2, SRSF1, RBM15, and CLK3) and protein synthesis (EIF3G, RPS6, TSC2, RPTOR, and EIF4B) pathways. Collectively, these results show that LIMK2 inhibition led to decreased phosphorylation for a wide-array of proteins associated with many different biological processes. This, in turn, may affect the activity and/or stability of these proteins and thereby regulate the ability of LIMK2 to facilitate the metastatic attributes of TNBC cells. Taken together, we have created a comprehensive catalog of putative LIMK2 targets in TNBC cells. Further detailed characterization of these targets will likely reveal new functions of LIMK2 in TNBC, other subtypes of breast cancer, and other cancer types.

Among the proteins identified in our SILAC analysis, we decided to further investigate SRPK1 as a mediator of LIMK2 function. SRPK1 is a serine/arginine protein kinase that specifically phosphorylates its substrates at serine residues in regions rich in arginine/serine dipeptides (known as RS domains) and is necessary for phosphorylation of SR splicing factors and the regulation of splicing^8,45–47^. Additionally, SRPK1 exerts metastasis-promoting activities in a wide variety of cancers, including breast cancer^32,34–37^. It is overexpressed in a variety of cancers and promotes tumor and metastatic growth^39,48–50^. Another recent study showed that SRPK1 maintains acute myeloid leukemia through effects on the isoform usage of epigenetic regulators including BRD4^51^.

We showed that like LIMK2, SRPK1 is overexpressed in several breast cancer subtypes compared to normal breast tissue. We also found that similar to LIMK2, SRPK1 was overexpressed in TNBC compared to other breast cancer subtypes, and SRPK1 overexpression in breast cancer predicted increased incidence of cancer metastasis, recurrence, and death. Our SILAC study identified reduced SRPK1 phosphorylation at five sites (Ser7, Ser9, Ser51, Ser309, and Ser311) after LIMK2 inhibition. Of these sites, Ser51 was previously shown to be phosphorylated by casein kinase 2^52^. Additional experiments revealed that the genetic and pharmacological inhibition of SPRK1 phenocopied the effects of LIMK2 inhibition, indicating that this kinase, in addition to other LIMK2 substrates, could be an important downstream mediator of LIMK2 function. Because LIMK2 was overexpressed in TNBC and in vivo studies showed that its pharmacological inhibition can block the metastatic attributes of TNBC cells, we also tested the LIMK2 small-molecule inhibitor LX7101^30^ in a mouse model of spontaneous TNBC metastasis. We found that the LIMK2 inhibitor LX7101 blocked metastatic progression without significantly affecting primary tumor growth, indicating that LIMK2 is important in the distal metastatic progression of TNBC cells. Collectively, our data link LIMK2 overexpression to TNBC metastasis and identify LIMK2 as a potential drug target for treating TNBC through its effects on SPRK1 and potentially other downstream targets identified by our SILAC studies.

## Materials and methods

### Cell culture conditions and reagents

Short tandem repeat (STR) profile verified MDA-MB-231, BT-549, and MDA-MB-468 cell lines were obtained from the American Type Culture Collection (ATCC, Manassas, VA, USA) and maintained as recommended by the ATCC. MDA-MB-231 and MDA-MB-468 cells were grown in Dulbecco’s Modified Eagle Medium (DMEM; Life Technologies, Thermo Fisher Scientific, Waltham, MA, USA), supplemented with 10% fetal bovine serum (FBS; Life Technologies, Thermo Fisher Scientific) and 1% penicillin/streptomycin (Life Technologies) under 5% CO_2_. BT-549 cells were grown in Roswell Park Memorial Institute (RPMI) 1640 medium (Life Technologies, Thermo Fisher Scientific) supplemented with 10% FBS and 1% penicillin/streptomycin in 5% CO_2_. Mycoplasma negative status for all cell lines was verified using MycoAlert mycoplasma detection kit (Lonza), and were routinely tested for the lack of mycoplasma contamination.

### Cell labeling and SILAC analysis

Cells were seeded at 15% confluency in the respective complete medium (for MDA-MB-231: RPMI + 10% dialyzed FBS + 1% Pen-Strep). The labeling medium was deficient in lysine and arginine and supplemented with light- or heavy-labeled lysine (^13^C_6_^15^N_2_) and light- or heavy-labeled arginine (^13^C_6_^15^N_4_). Cells were subsequently cultured for at least five doublings in light or heavy medium and achieved over 95% labeling efficiency in our pilot experiments. After labeling, cells were treated for 6 hr with 5 μM LIMK2 inhibitor LX7101. After treatment, cells were trypsinized and counted to obtain a cell pellet of 2 × 10^7^ cells/condition and subjected to SILAC analysis using mass spectrometry. Additional details are presented in the Supplemental Materials and Methods section.

### SILAC data analysis for identifying the preferred LIMK2 amino acid context for phosphorylation

To identify the LIMK2 phosphorylation consensus site from the SILAC data, we used a prediction algorithm developed in house. The motifs were generated by the R/Bioconductor package dagLogo (v.1.9.2). The background of the motifs was built from the human proteome retrieved via the R/Bioconductor package UniProt.ws (v.2.11.9). Lists of all quantified phosphopeptides for the MDA-MB-231 cell line are presented in Table S5. The SILAC proteomics data have been submitted to PRIDE (https://www.ebi.ac.uk/pride/archive/). The data can be accessed via the PRIDE database using the accession number PXD008246.

### Immunohistochemistry

Formalin-fixed, paraffin-embedded tissue microarray slides containing TNBC tissues were obtained from Yale Tissue Microarray Facility (Cat No. YTMA311 (*n* = 92), YTMA341 (*n* = 53), and YTMA347 (*n* = 54)) or from US Biomax, Inc. (Cat No. BC081120e). Briefly, following deparaffinization of slides, antigen retrieval was performed in citrate buffer (pH 6.0) at 97 °C for 20 min using the Lab Vision PT Module (Thermo Fisher Scientific). Endogenous peroxides were blocked using hydrogen peroxide, and proteins were blocked using 0.3% BSA. Slides were incubated in LIMK2 antibody (dilution 1:50) or SRPK1 antibody (dilution 1:500) followed by secondary anti-rabbit HRP-conjugated antibody (Dako, CA, USA). Slides were stained using the liquid DAB+substrate chromogen system (Dako) and counterstained using automation hematoxylin histological staining reagent (Dako). LIMK2 staining of tissue microarray slides was independently scored by three board-certified breast pathologists, Drs. Malini Harigopal, Ester Yoon, and Padmini Manrai, who were blinded to the slide identities. SPRK1 scoring was performed by Drs. Malini Harigopal and Kamalgeet Singh. The criteria for scoring IHC staining included percentage of positively stained cells and intensity of observed staining^53^. For the purpose of our study, LIMK2 expression was recorded using an arbitrary scale based on the percentage of positive tumor cells: 0–25%; 26–50%; 51–75%, and 76–100%.

To validate the specificity of the LIMK2 antibody used for IHC, immunofluorescence staining was performed on MDA-MB-231 cells expressing *LIMK2* shRNAs or a negative control shRNA. Antibody specificity was also validated separately by immunoblot analysis. Details of the antibodies used for IHC analyses are listed in Table S8.

### Mouse tumorigenesis and spontaneous metastasis experiment with LX7101 treatment

MDA-MB-231 cells stably expressing firefly luciferase under the control of a cytomegalovirus promoter were generated by cotransfection of the transposon vector piggyBac GFP-Luc and the helper plasmid Act-PBase as described previously^54^. Cells with stable transposon integration were selected using blasticidin S (Thermo Fisher Scientific). MDA-MB-231-GFP-*F-Luc* cells (10 × 10^6^) in Matrigel (Corning; Cat No. 356237) were then injected subcutaneously into four female NSG mice aged 5–6 weeks per experimental group (Jackson Laboratory, Stock No. 005557). Tumor volume was measured every week. Tumor size was calculated using the following formula: length × width^2^ × 0.5. The vehicle (0.5% methyl cellulose in phosphate-buffered saline [PBS]) or LX7101 (50 mg/kg body weight) was administered by oral gavage every alternate day starting week 4 of injecting the cells (tumor volumes ∼80–100 mm^3^) until the end of experimental period. At the end of the experiment (4 weeks after the treatment), the mice were killed, and the lungs were imaged using the IVIS Spectrum in vivo imaging system (Perkin Elmer). All protocols for mouse experiments were approved by the Institutional Animal Care and Use Committee of the University of Alabama at Birmingham.

### Statistical analysis

All of the experiments were conducted in triplicate. The results of the individual experiments are expressed as the means ± SD. For measuring statistical significance, the *P*-values were calculated using the two-tailed unpaired Student’s *t* test in GraphPad Prism version 8.0 for Macintosh (GraphPad Software, San Diego, California, USA). Unpaired *t*-test with Welch’s correction was applied to data with unequal variance. Contingency analysis was performed using Fisher’s exact test in GraphPad Prism version 8.0 for Macintosh (GraphPad Software, San Diego, California, USA).

## Supplementary information

Supplemental Information

Table S1

Table S2

Table S3

Table S4

Table S5

Table S6

Table S7

Table S8
