# Supplementary material for: LIMK2 promotes the metastatic progression of triple-negative breast cancer by activating SRPK1
Source: Oncogenesis. 2020 Aug 28;9(8):77. doi: 10.1038/s41389-020-00263-1 (PMC7455732; doi:10.1038/s41389-020-00263-1)
Supplement: Supplementary file 1 — Supplemental Information [file 41389_2020_263_MOESM1_ESM.pdf]

SUPPLEMENTAL INFORMATION

SUPPLELENTARY FIGURES AND FIGURE LEGENDS

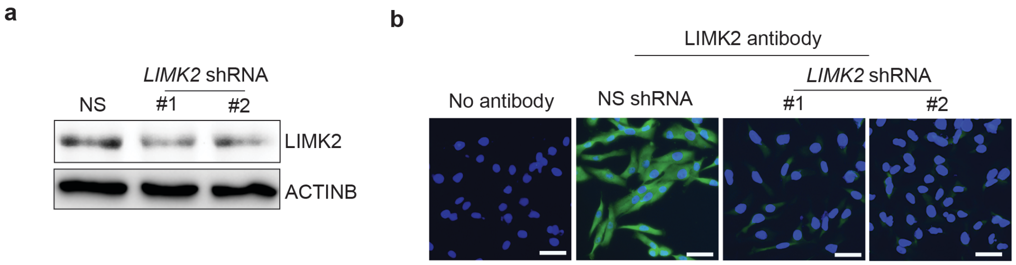

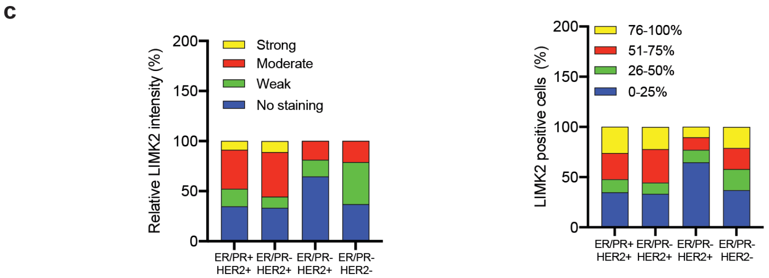

Subtype	No staining	Weak	Moderate	Strong
ER/PR+, HER2+	34.78	17.39	39.13	8.70
ER/PR-, HER2+	33.33	11.11	44.44	11.11
ER/PR+, HER2-	64.58	16.67	18.75	0.00
ER/PR-, HER2-	36.84	42.11	21.05	0.00

Subtype	0-25%	26-50%	51-75%	76-100%
ER/PR+, HER2+	34.78	13.04	26.09	26.09
ER/PR-, HER2+	33.33	11.11	33.33	22.22
ER/PR+, HER2-	64.58	12.50	12.50	10.42
ER/PR-, HER2-	36.84	21.05	21.05	21.05

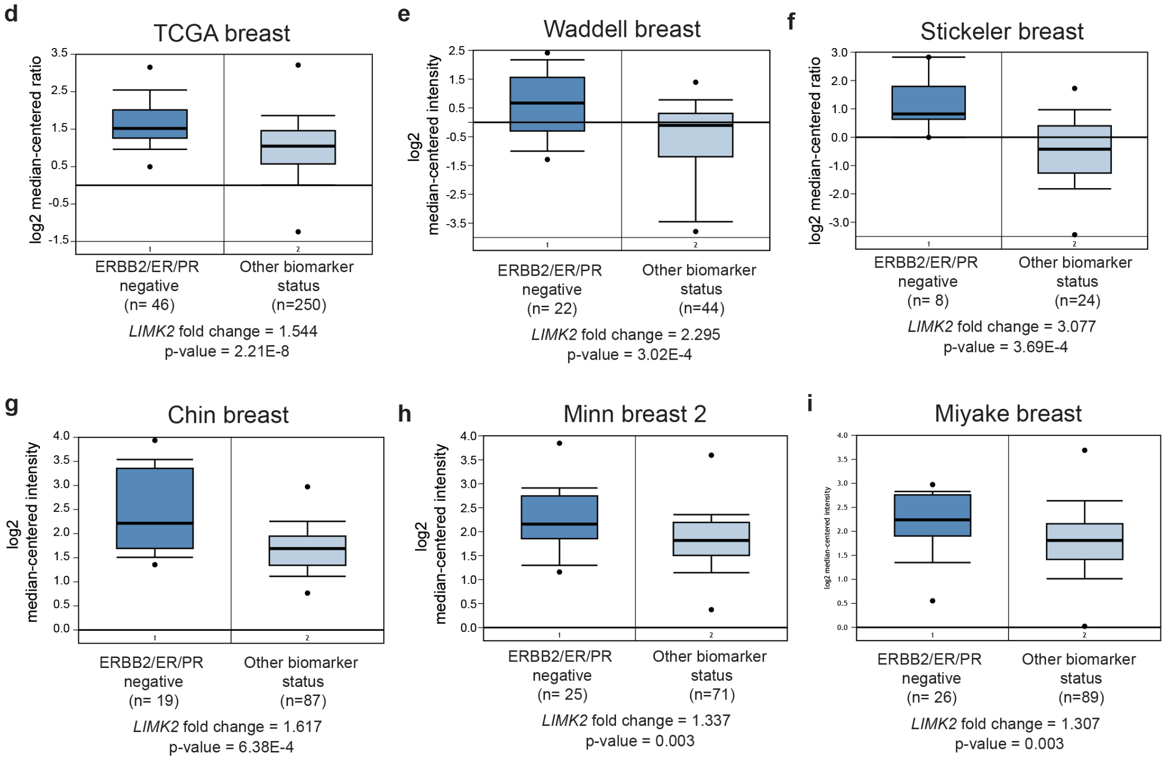

Fig. S1. LIMK2 is overexpressed in TNBC. a. Validation of the specificity of the LIMK2 antibody used for immunohistochemistry by immunoblot by analyzing MDA-MB-231 cells expressing either *LIMK2* shRNAs or non-specific (NS) shRNA. **b.** Validation of the specificity of the LIMK2 antibody used for immunohistochemistry by immunofluorescence in MDA-MB-231 cells expressing either *LIMK2* shRNAs or non-specific (NS) shRNA using DAPI (blue)/LIMK2 (green) immunofluorescence and confocal microscopy. Scale bar, 100 μm. **c.** LIMK2 expression in indicated breast cancer subtype in the tissue microarray (TMA) from US Biomax (BC081120e). **d.** TCGA breast dataset was analyzed for *LIMK2* mRNA expression. Average fold change in *LIMK2* expression in patient-derived TNBC samples relative to the breast cancer samples expressing other biomarkers is shown. **e.** Waddell breast dataset was analyzed for *LIMK2* mRNA expression. Average fold change in *LIMK2* expression in patient-derived TNBC samples relative to the breast cancer samples expressing other biomarkers is shown. **f.** Stickeler breast dataset was analyzed for *LIMK2* mRNA expression. Average fold change in *LIMK2* expression in patient-derived TNBC samples relative to the breast cancer samples expressing other biomarkers is shown. **g.** Chin breast dataset was analyzed for *LIMK2* mRNA expression. Average fold change in *LIMK2* expression in patient-derived TNBC samples relative to the breast cancer samples expressing other biomarkers is shown. **h.** Minn breast 2 dataset was analyzed for *LIMK2* mRNA expression. Average fold change in *LIMK2* expression in patient-derived TNBC samples relative to the breast cancer samples expressing other biomarkers is shown. **i.** Miyake breast dataset was analyzed for *LIMK2* mRNA expression. Average fold change in *LIMK2* expression in patient-derived TNBC samples relative to the breast cancer samples expressing other biomarkers is shown.

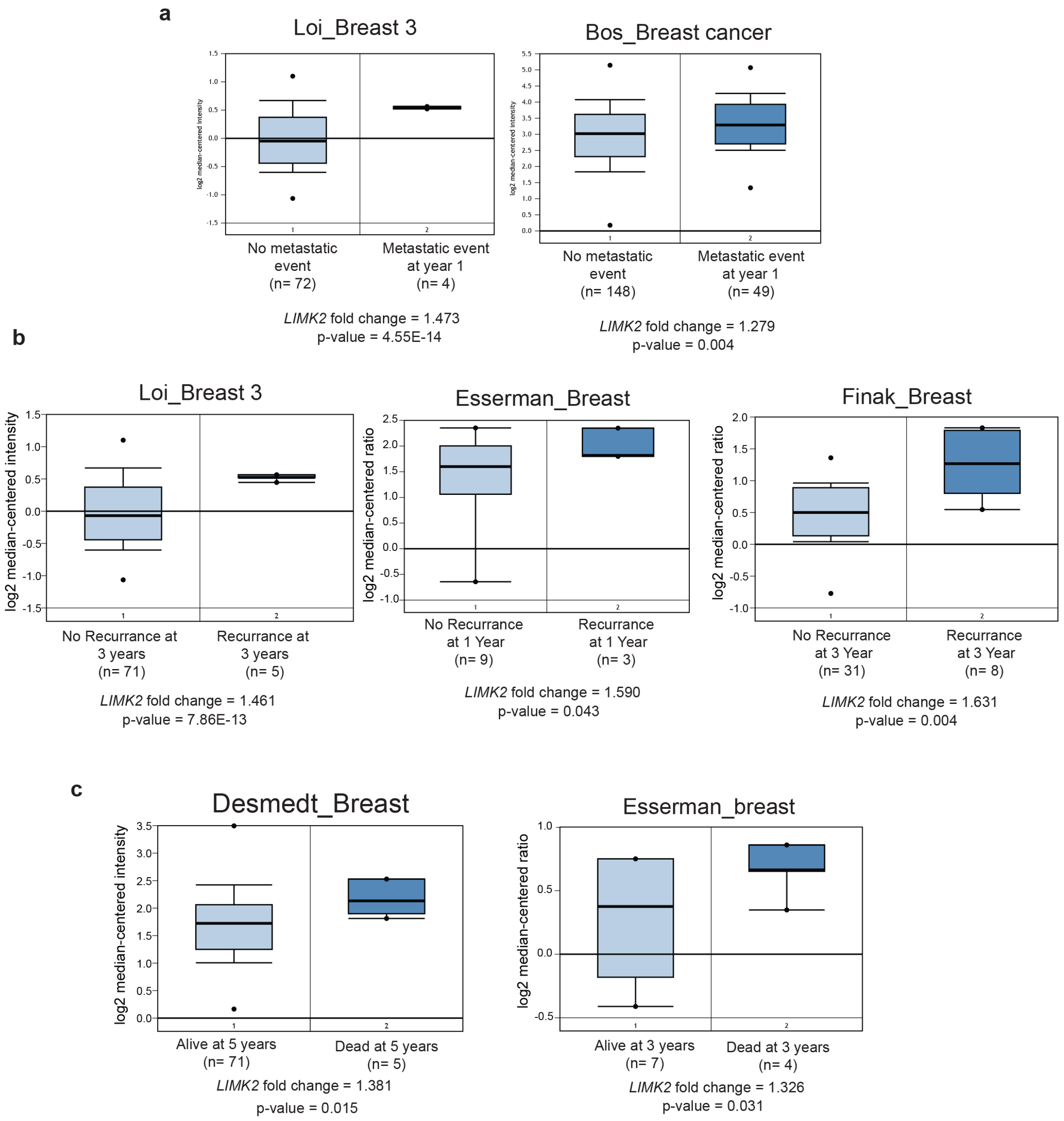

Fig. S2. Higher LIMK2 expression is associated with increased metastasis, increased rate of recurrence and increased incidences of death in breast cancer patients. a. Comparison of *LIMK2* expression in patient-derived breast cancer samples from subjects with no metastatic event or with metastatic event at 1 year. *P*-value for the indicated comparison is shown. **b.** Comparison of *LIMK2* expression in patient-derived breast cancer samples from subjects with no recurrence or recurrence at 3 or 1 years. *P*-value for the indicated comparison is shown. **c.** Significant differences in *LIMK2* mRNA expression for patient-derived breast cancer samples from patients who were alive or dead at 3 or 5 years; *P*-values for the indicated comparisons are shown.

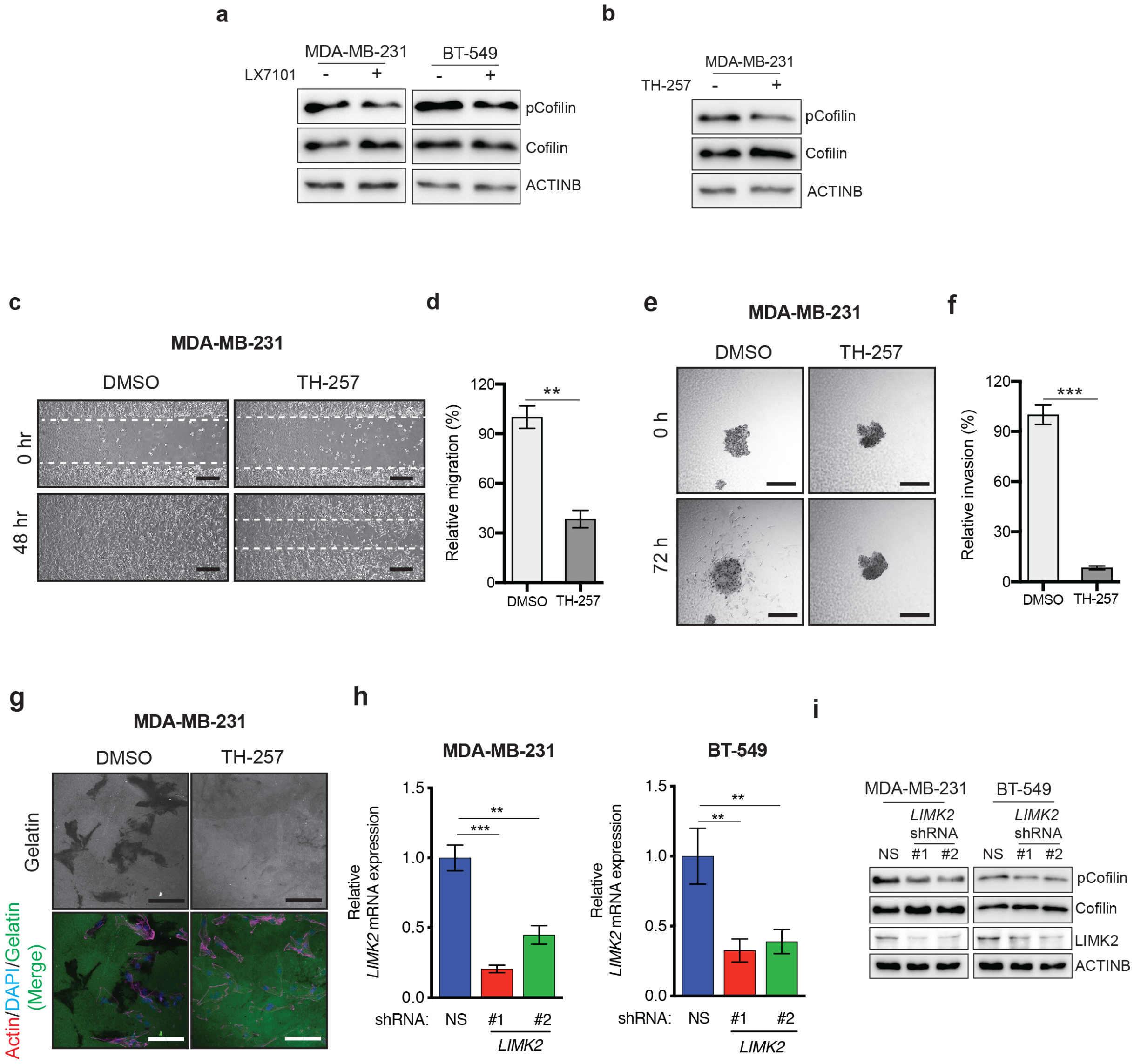

Fig. S3. Effect of LIMK2 inhibitor treatment on the metastatic properties of TNBC cells, and knockdown validation of LIMK2 in cells expressing *LIMK2* shRNAs. a. MDA-MB-231 and BT-549 cells were treated with the vehicle or LX7101 (5 µM) for 6 hr. Phosphorylation of cofilin was analyzed using immunoblotting. ACTINB was used as a loading control. **b.** MDA-MB-231 cells were treated with 10 µM LIMK2 inhibitor (TH-257) for 6 hr. Phosphorylation of cofilin was analyzed using immunoblotting. ACTINB was used as a loading control. **c.** MDA-MB-231 cells untreated or treated with LIMK2 inhibitor (TH-257) were analyzed using a wound healing assay. Representative images at the indicated times are shown. Scale bar, 250 µm. **d.** Relative migration (%) calculated from the data presented in panel c. **e.** MDA-MB-231 cells untreated or treated with LIMK2 inhibitor were analyzed using the 3D spheroid invasion assay. Representative images at the indicated times are shown. Scale bar, 250 µm. **f.** Relative invasion (%) calculated from the data presented in panel e. **g.** Extracellular matrix degradation capacity of MDA-MB-231 cells untreated or treated with LIMK2 inhibitor was analyzed using the gelatin degradation assay. Cells were stained with phalloidin (red) and DAPI (blue). Gelatin (green) degradation appears as black areas.

Representative images are shown. Scale bar, 500 µm. **h.** MDA-MB-231 and BT-549 cells expressing *LIMK2* shRNA were analyzed for cofilin phosphorylation using immunoblotting. ACTINB was used as a loading control. **i.** MDA-MB-231 and BT-549 cells expressing either nonsilencing (NS) or *LIMK2* shRNA were analyzed for the expression of *LIMK2* mRNA using RT-qPCR. *LIMK2* mRNA expression relative to NS shRNA–expressing cells is shown. Data are represented as the means ± SD. $^{**}P < 0.01$; and $^{***}P < 0.001$.

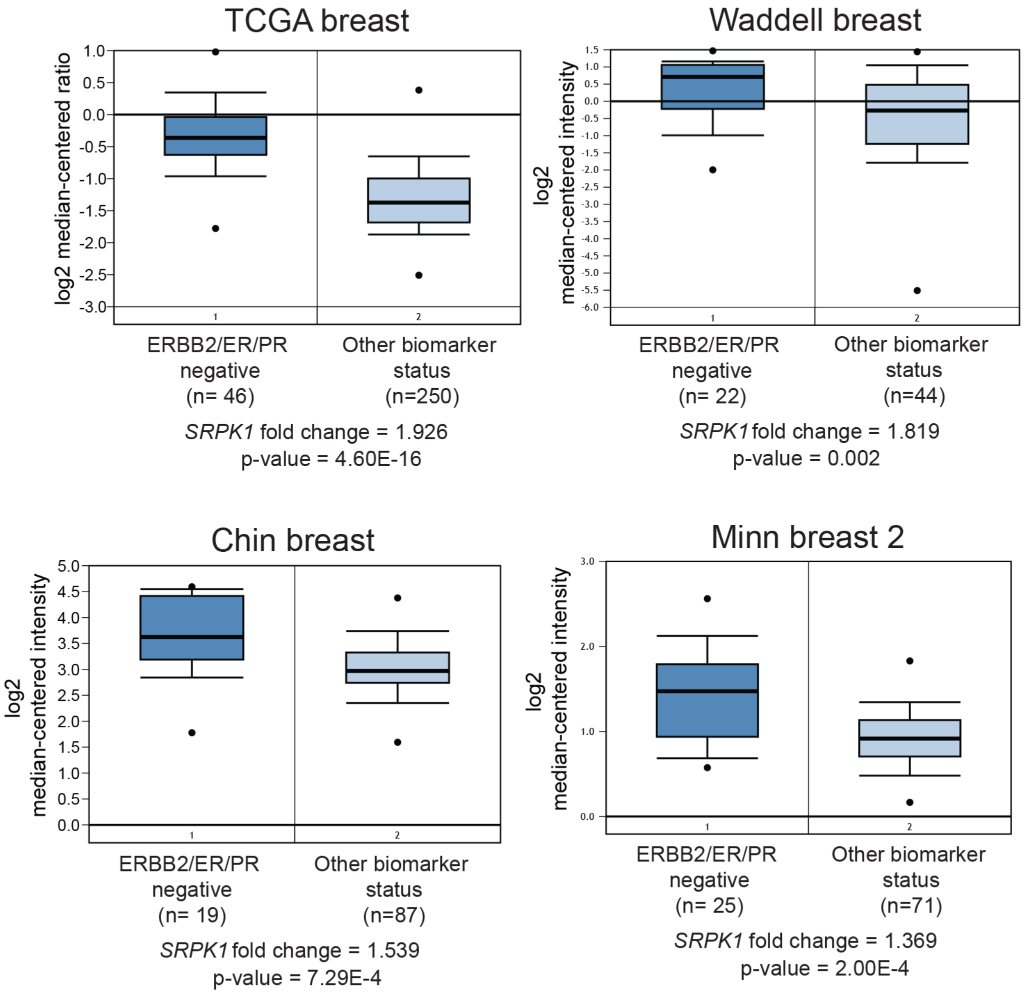

Fig. S4. SRPK1 is overexpressed in patient-derived TNBC samples. Indicated breast cancer datasets were analyzed for *SRPK1* mRNA expression. Average fold change in *SRPK1* expression in patient-derived TNBC samples relative to the breast cancer samples expressing other biomarkers is shown. p-values are also shown.

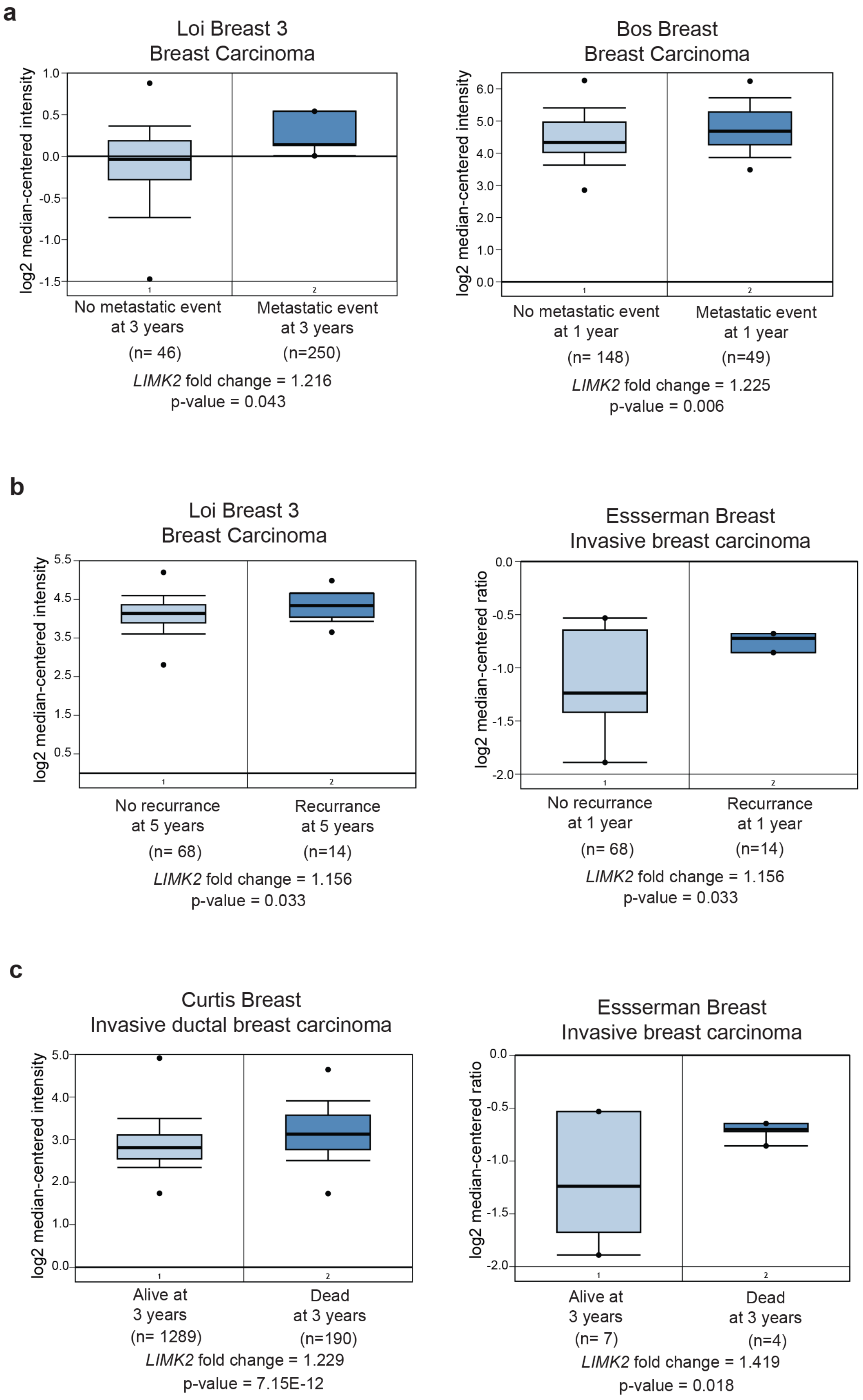
a
Loi Breast 3
Breast Carcinoma
log2 median-centered intensity
1.0
0.5
0.0
-0.5
-1.0
-1.5
1
2
No metastatic event
at 3 years
(n= 46)
Metastatic event
at 3 years
(n=250)
LIMK2 fold change = 1.216
p-value = 0.043
Bos Breast
Breast Carcinoma
log2 median-centered intensity
6.0
5.0
4.0
3.0
2.0
1.0
0.0
1
2
No metastatic event
at 1 year
(n= 148)
Metastatic event
at 1 year
(n=49)
LIMK2 fold change = 1.225
p-value = 0.006
b
Loi Breast 3
Breast Carcinoma
log2 median-centered intensity
5.5
4.5
3.5
2.5
1.5
0.5
1
2
No recurrance
at 5 years
(n= 68)
Recurrance
at 5 years
(n=14)
LIMK2 fold change = 1.156
p-value = 0.033
Essserman Breast
Invasive breast carcinoma
log2 median-centered ratio
0.0
-0.5
-1.0
-1.5
-2.0
1
2
No recurrance
at 1 year
(n= 68)
Recurrance
at 1 year
(n=14)
LIMK2 fold change = 1.156
p-value = 0.033
c
Curtis Breast
Invasive ductal breast carcinoma
log2 median-centered intensity
5.0
4.0
3.0
2.0
1.0
0.0
1
2
Alive at
3 years
(n= 1289)
Dead
at 3 years
(n=190)
LIMK2 fold change = 1.229
p-value = 7.15E-12
Essserman Breast
Invasive breast carcinoma
log2 median-centered ratio
0.0
-0.5
-1.0
-1.5
-2.0
1
2
Alive at
3 years
(n= 7)
Dead
at 3 years
(n=4)
LIMK2 fold change = 1.419
p-value = 0.018

Fig. S5. Higher SRPK1 expression is associated with increased metastasis, increased rate of recurrence and increased incidences of death in breast cancer patients. a. Comparison of *SRPK1* expression in indicated breast cancer dataset with subjects with no metastatic event or with metastatic event at indicated times. Fold changes and *p*-values for the comparisons are shown. for the indicated comparison is shown. **b.** Comparison of *SRPK1* expression in patient-derived breast cancer samples from subjects with no recurrence or recurrence at indicated times. Fold changes and *p*-values for the comparison are shown **c.** Comparison of *SRPK1* expression in patient-derived breast cancer samples from subjects that were alive or dead at indicated times. Fold changes and *p*-values for the comparison are shown.

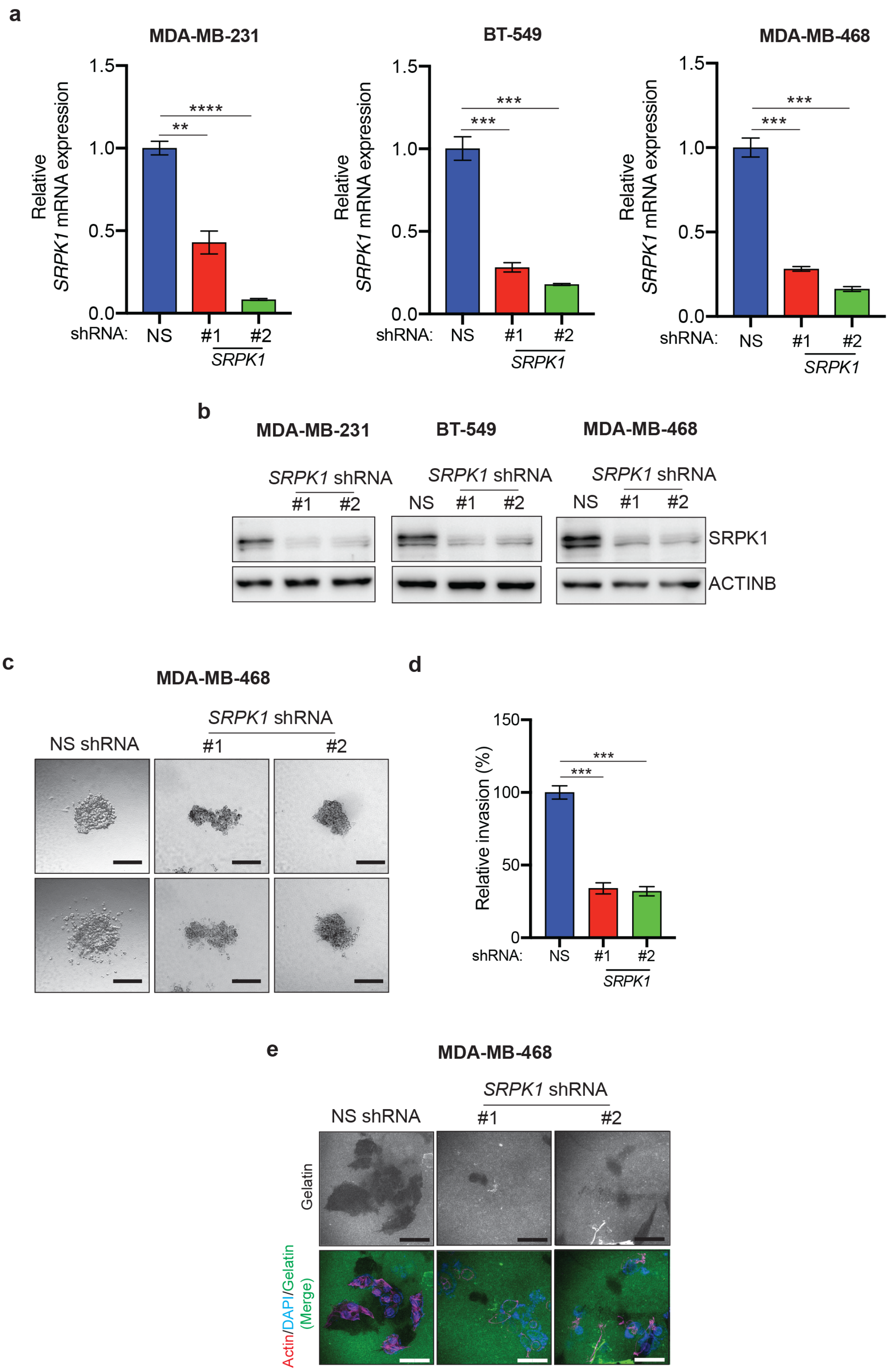
a
MDA-MB-231
BT-549
MDA-MB-468
Relative SRPK1 mRNA expression
1.5
1.0
0.5
0.0
****
**
***
shRNA:
NS
#1
#2
SRPK1
b
SRPK1 shRNA
SRPK1
ACTINB
c
NS shRNA
d
150
100
50
0
Relative invasion (%)
e
Gelatin
Actin/DAPI/Gelatin (Merge)

Fig. S6. Knockdown validation of *SRPK1* in cells expressing *SRPK1* shRNAs. a. TNBC cell lines (MDA-MB-231, BT-549, and MDA-MB-468) expressing either nonsilencing (NS) or *SRPK1* shRNA were analyzed for the expression of *SRPK1* mRNA using RT-qPCR. *SRPK1* mRNA expression relative to NS shRNA–expressing cells is shown. **b.** TNBC cell lines (MDA-MB-231, BT-549, and MDA-MB-468) expressing *SRPK1* shRNA were analyzed for SRPK1 expression by immunoblotting. ACTINB was used a loading control. **c.** MDA-MB-468 cells expressing *SRPK1* shRNA were analyzed using a 3D spheroid invasion assay. Representative images at the indicated times are shown. Scale bar, 250 µm. **d.** Relative invasion (%) calculated from the data presented in panel c. **e.** Extracellular matrix degradation capacity of MDA-MB-468 cells expressing *SRPK1* shRNA was analyzed using the gelatin degradation assay. Cells were stained with phalloidin (red) and DAPI (blue). Gelatin (green) degradation appears as black areas. Representative images are shown. Scale bar, 500 µm. Data are represented as the means ± SD. $^{**}P < 0.01$; $^{***}P < 0.001$; and $^{****}P < 0.0001$.

SUPPLEMENTARY TABLE CAPTIONS

Table S1. Summary of immunohistochemistry staining for LIMK2 in various human-derived breast cancer subtypes (including triple-negative breast cancer) and normal breast tissues in tissue microarrays from US Biomax.

Table S2. Summary of immunohistochemistry staining for LIMK2 in human-derived triple-negative breast cancer and normal breast tissues in tissue microarrays from Yale Tissue Microarray Facility.

Table S3. List of SILAC-identified proteins whose phosphorylation was downregulated upon LIMK2 inhibition in MDA-MB-231 cells.

Table S4. Analysis of the SILAC data using ingenuity pathway analysis (IPA) of proteins with reduced phosphorylation upon LIMK2 inhibition in MDA-MB-231 cells.

Table S5. Lists of all quantified phosphopeptides from SILAC analysis of LX7101 treated MDA-MB-231 cells.

Table S6. Summary of immunohistochemistry staining for SRPK1 in various human-derived breast cancer subtypes (including triple-negative breast cancer) and normal breast tissues in tissue microarrays from US Biomax.

Table S7. Summary of immunohistochemistry staining for SRPK1 in human-derived triple-negative breast cancer and normal breast tissues in tissue microarrays from Yale Tissue Microarray Facility.

Table S8. Reagents and resources used in this study.

SUPPLEMENTAL METHODS

RNA preparation, cDNA synthesis, and RT-qPCR analysis

Total RNA was extracted using TRIzol (Invitrogen) and purified using the RNeasy Mini Kit (Qiagen) according to the manufacturer's instructions. cDNA was generated using a ProtoScript first-strand cDNA synthesis kit (New England Biolabs). Quantitative PCR was performed using Power SYBR Green Master Mix (Life Technologies). The oligonucleotide sequences used for RT-qPCR are provided in **Table S8**.

shRNAs, transfection, lentivirus or retrovirus preparation, and stable cell line generation

All shRNAs for LIMK2 and SRPK1 were obtained from Open Biosystems and are listed in the **Table S8**. Lentiviral particles carrying shRNA were generated by cotransfecting shRNA plasmids with the lentiviral packaging plasmids pSPAX2 and pMD2.G into 293T cells using Effectene (Qiagen) according to the manufacturer's instructions. Culture medium was filtered using a 0.45-μm sterile filter to remove any dead or live cells and from lentiviral particles. Stable cell lines were generated by infecting various TNBC cell lines with shRNA lentivirus in 12-well plates followed by puromycin selection (0.5–0.75 μg/ml).

Immunoblotting

Immunoblot analysis was performed, as described previously [1]. Briefly, protein extracts were prepared in ice-cold immunoprecipitation (IP) lysis buffer (Thermo Scientific, IL, USA) containing protease inhibitor (Roche, USA) and phosphatase inhibitor cocktail (Sigma-Aldrich, USA). Lysed samples were centrifuged at 12000 rpm for 40 min, and clarified supernatants were stored at −80°C. Equal amounts of protein samples were electrophoresed on 6–12% sodium

dodecyl sulfate–polyacrylamide gels and transferred onto polyvinylidene difluoride membranes (Millipore, MA, USA). The membranes were blocked and probed with primary antibodies. After washing, the membranes were incubated with appropriate horseradish peroxidase (HRP)–conjugated secondary antibodies (1:2000) (GE Healthcare Bio-Sciences, MA, USA), and the blots were developed using luminescence detection reagents (Thermo Scientific). The antibodies used for immunoblotting are listed in **Table S8**.

Immunofluorescence staining

MDA-MB-213 and BT-549 cells were plated onto coverslips in multi-well chamber slides. After 24 hr, these cells were washed with PBS and fixed with 3.7 % paraformaldehyde. Cells were then permeabilized using 0.3% Triton X-100. After washing with PBS, blocking was performed using 5% bovine serum albumin (BSA) in PBS. Then, cells were probed with vinculin primary antibody in 5% BSA in PBS (1:200) for 2 hr at room temperature. After washing, the cells were incubated with Alexa488-conjugated anti-mouse secondary antibody (1:1000) diluted in PBS with 5% BSA in PBS for 1 hr at room temperature. Then, cells were stained with AlexaFluor 594 phalloidin for actin visualization and mounted onto microscopic glass slides. Fluorescence images were acquired using a LEICA SP5 confocal laser scanning microscope. Antibody information is provided in the **Table S8.**

Wound healing assay

For the wound healing assay, MDA-MD-231 and BT-549 cells were grown in 12-well plates until fully confluent. A scratch was created using a sterile 20-μl pipette tip, and cell

migration was monitored at 0, 12, and 24 hr using light microscopy. The quantification of wound healing was performed using ImageJ software (https://imagej.nih.gov/ij/).

Gelatin degradation assay

The gelatin degradation assay was performed as per the manufacturers' instructions (QCMTM Gelatin Invadopodia Assay, Millipore). In brief, glass coverslips were washed and sterilized in 70% ethanol, coated with poly-L-lysine and fixed with glutaraldehyde. After fixation, the coverslips were washed with PBS and coated with mixture of fluorescein isothiocyanate–conjugated collagen. The coated coverslips were sterilized in 70% ethanol and residual free aldehydes were quenched by incubation in growth medium for 30 min. After quenching, breast cancer cells were plated on these coverslips for 24 hr to allow cells to degrade the matrix. The cells were then fixed with 3.7% paraformaldehyde and stained with AlexaFluor 594 phalloidin and 4′,6-diamidino-2-phenylindole (DAPI). Gelatin degradation was analyzed using a LEICA SP5 confocal laser scanning microscope.

In vitro kinase assay

To confirm if LIMK2 phosphorylates SRPK1 protein, an *in vitro* kinase assay was performed as described previously [2]. Briefly, SRPK1 recombinant human protein (0.25 µg) was incubated with LIMK2 recombinant human protein and K buffer (25 mM HEPES pH 7.9, 5 mM $MgCl_2$, 0.1 mM EDTA) in the presence or absence of 1 mM ATP. The reaction mixture was incubated for 1 hr at 30 °C. The reaction was terminated by adding 4× SDS-PAGE loading buffer. The protein samples were separated by SDS–PAGE and immunoblotted (for phosphorylated-Serine and total

SRPK1), and the blots were visualized using luminescence detection reagents (Thermo Scientific). The antibodies used for immunoblotting are listed in **Table S8**.

3D spheroid invasion assay

The 3D spheroid invasion assay was performed as previously described [3]. In brief, TNBC cells were counted, and the concentration was adjusted to a density of 5×10^4 cells/mL. Twenty μl drops of cell suspension were placed on top of the tissue culture plate lid and inverted. Spheroids were allowed to form for 3–6 days depending on the cell line. Formed spheroids were embedded in collagen I matrix (2 mg/mL) and allowed to invade. The cell invasion was monitored at 0, 24, 48, and 72 hr using light microscopy.

Chemical Inhibitors

The LIMK2 inhibitor LX7101 was purchased from Cayman Chemicals. The LIMK2 inhibitor TH-257 was purchased from Sigma-Aldrich. The SRPK1 inhibitor SRPIN340 was obtained from Selleckchem. The treatment concentrations and time of treatment are described in the **Table S8** and relevant figure legends.

Sample preparation for SILAC analysis

The heavy and light cells pellets were lysed in radioimmunoprecipitation buffer spiked with protease and phosphatase inhibitors using short 15 sec sonication bursts. Lysates were centrifuged at 14,000 rpm for 20 min. After centrifugation, the supernatants were collected and protein concentration was measured using a Hitachi L-8900 amino acid analyzer. From each sample, 200 μg protein was aliquoted, combined, and precipitated using a methanol-chloroform

precipitation method. The protein pellets were resuspended in 8 M urea/0.4 M ammonium bicarbonate buffer, reduced with 45 mM DTT for 30 min at 37°C, alkylated with 100 mM iodoacetamide for 30 min in the dark at room temperature, and digested with Lys-C protease (1:20 w/w) by incubating overnight (~16 hr) at 37°C. The Lys-C digest was further diluted and digested with trypsin (1:20 w/w) by incubating for 8 hr at 37°C. The digest was desalted with MacroSpin column (The Nest Group, Inc., Southboro, MA) and dried down in a SpeedVac concentrator. Desalted peptides were then enriched with phosphopeptides using titanium dioxide resin imbedded in 10-µL tips (Glygen Corp., Columbia, MD). Flow-throughs were reserved and enriched peptides were eluted using a 1:33 ratio of ammonium hydroxide to water. The SpeedVac-dried flow-through and elution fractions were resuspended in buffer A (0.1% formic acid in water) and subjected to liquid chromatography-tandem mass spectrometry (LC-MS/MS) analysis.

Mass spectrometry data acquisition and analysis

Samples were analyzed by LC-MS/MS on an Orbitrap Fusion Tribrid mass spectrometer (Thermo Scientific, San Jose, CA) interfaced with a nanoACQUITY UPLC system (Waters, Milford, MA) at the front end. Samples were loaded into a trapping column (nanoACQUITY UPLC Symmetry C18 Trap Column; 180 µm × 20 mm; No. 186006527) at a flowrate of 5 µl/min and separated with a C18 column (nanoACQUITY Column Peptide BEH C18; 75 µm × 250 mm; No. 186003545). The peptides were eluted with buffer B (0.1% formic acid in acetonitrile) in a gradient from 6% to 35% in 150 min at a flowrate of 300 nL/min. LC-MS/MS data were acquired using 3 sec, the top speed data-dependent acquisition mode.

Peptides and proteins were identified and quantified with the Sequest HT search engine using Proteome Discoverer version 2.1 (Thermo Fisher Scientific) software. A standardized

SILAC 2plex (Arg10, Lys8) quantification workflow in the Proteome Discoverer was slightly modified as described below and used for analysis. Briefly, LC-MS/MS data were searched against the SwissProt human database (downloaded in September 2015; number of protein entries: 20,193). The search criteria included 10 ppm precursor mass tolerance, 0.02 Da fragment mass tolerance, and a trypsin miscleavage setting of two. Static modification settings included carbamidomethylation (+57.021 Da) on cysteine, whereas dynamic modifications were set to include oxidation (+15.995 Da) on methionine and phosphorylation (+79.966 Da) on serine, threonine, and tyrosine. Peptide spectrum matches were verified based on q-values set to a 1% false discovery rate using Percolator. The Precursor Ions Quantifier node was used in the processing step workflow, and the Peptide and Protein Quantifier node was selected for the consensus workflow to calculate and quantify peptides, protein abundances, and ratios.

Bioinformatic analysis of breast cancer datasets

Breast cancer datasets were downloaded from Oncomine (https://www.oncomine.org), analyzed for *LIMK2* expression, and graphed as box plots to compare TNBC samples with the breast cancer samples expressing other biomarkers [4-9].. The TCGA Breast Dataset analyzed 532 invasive breast carcinoma, 61 paired normal breast tissue and 3 paired metastatic samples. Sample data included age, histology, TNM stage, ER/PR/ERBB2 status, sex, stage, and others. This dataset consisted of Level 2 (processed) data from the TCGA data portal. The Waddell Breast Dataset analyzed 85 familial breast carcinoma samples from 76 patients. Sample data included BRCA1, BRCA2, and ATM mutation status, ER/PR/ERBB2 status, and others using Illumina DASL HumanRef-8 Whole Genome v3.0 array. The Stickeler Breast Dataset analyzed 57 breast carcinoma samples were. Sample data included patient treatment response, treatment, T and N

stage, grade, and others using Agilent Human Genome 44K array. The Chin Breast Dataset analyzed 118 frozen primary breast tumors on Affymetrix U133A microarrays. Sample data included stage, age, ERBB2 status, estrogen receptor status, lymph node status, p53 status, progesterone receptor status, race, and others. The Minn Breast 2 Dataset included microarray data (HG-U133A), clinical data (age, tumor size, lymph node status, lung metastasis, bone metastasis, etc.), and molecular data (ER status, Path PR status, Her2 status, etc.) available from 99 primary breast cancer tumors. The Miyake Breast Dataset analyzed 115 breast carcinoma samples. Sample data included age, stage, grade, estrogen receptor status, progesterone receptor status, ERBB2 status and patient treatment response using Human Genome U133 Plus 2.0 Array.

The Loi Breast 3 Dataset analyzed 77 estrogen-receptor positive tamoxifen-treated primary breast cancer samples on Affymetrix U133 Plus 2.0 microarrays. Sample data included age, Elston grade, tumor size, progesterone receptor status, N stage, recurrence, and metastatic event status [10]. The Bos Breast Dataset analyzed 204 breast carcinoma samples on Human Genome U133 Plus 2.0 Array. Sample data included metastatic event status and N stage. The Esserman Breast Dataset analyzed 129 breast carcinoma samples and one normal breast sample on Agilent Human Genome 44K Array. Sample data included patient treatment response, estrogen receptor, progesterone receptor, ERBB2 status, survival, recurrence, and others [11]. The Finak Breast Dataset analyzed 53 breast tumor stroma samples and 6 normal breast stroma samples on Agilent 44K microarrays. Sample data included outcome, recurrence, grade, ER status, PR status, ERBB2 status, lymph node status, age, tumor size, and others [12]. The Desmedt Breast Dataset analyzed Gene expression levels of 198 breast cancer samples on Affymetrix U133A microarrays. Sample data included age, tumor size, surgery type, histopathology, angioinvasion, lymphocyte infiltrate, grade, estrogen receptor status, disease free survival, distant metastasis-free survival, time to

distant metastasis, clinical risk, and other clinical data [13]. The Curtis Breast Dataset analyzed 1,992 breast carcinoma samples and 144 paired normal breast samples for the METABRIC project on Illumina HumanHT-12 V3.0 R2 Array. Sample data included ER/PR/ERBB2 status, overall survival status and follow-up time, stage, grade, and others [14].

Supplemental References

1 Santra MK, Wajapeyee N, Green MR. F-box protein FBXO31 mediates cyclin D1 degradation to induce G1 arrest after DNA damage. *Nature* 2009; 459: 722-725.

2 Lee SE, Elphick LM, Anderson AA, Bonnac L, Child ES, Mann DJ *et al*. Synthesis and reactivity of novel gamma-phosphate modified ATP analogues. *Bioorg Med Chem Lett* 2009; 19: 3804-3807.

3 Vinci M, Box C, Eccles SA. Three-dimensional (3D) tumor spheroid invasion assay. *J Vis Exp* 2015: e52686.

4 Atlas) TTCG. The Cancer Genome Atlas - Invasive Breast Carcinoma Gene Expression Data.

5 Waddell N, Cocciardi S, Johnson J, Healey S, Marsh A, Riley J *et al*. Gene expression profiling of formalin-fixed, paraffin-embedded familial breast tumours using the whole genome-DASL assay. *J Pathol* 2010; 221: 452-461.

6 Stickeler E, Pils D, Klar M, Orlowsk-Volk M, Zur Hausen A, Jager M *et al*. Basal-like molecular subtype and HER4 up-regulation and response to neoadjuvant chemotherapy in breast cancer. *Oncol Rep* 2011; 26: 1037-1045.

7 Chin K, DeVries S, Fridlyand J, Spellman PT, Roydasgupta R, Kuo WL *et al*. Genomic and transcriptional aberrations linked to breast cancer pathophysiologies. *Cancer Cell* 2006; 10: 529-541.

8 Minn AJ, Gupta GP, Siegel PM, Bos PD, Shu W, Giri DD *et al*. Genes that mediate breast cancer metastasis to lung. *Nature* 2005; 436: 518-524.

9 Miyake T, Nakayama T, Naoi Y, Yamamoto N, Otani Y, Kim SJ *et al*. GSTP1 expression predicts poor pathological complete response to neoadjuvant chemotherapy in ER-negative breast cancer. *Cancer Sci* 2012; 103: 913-920.

10 Loi S, Haibe-Kains B, Desmedt C, Wirapati P, Lallemand F, Tutt AM *et al*. Predicting prognosis using molecular profiling in estrogen receptor-positive breast cancer treated with tamoxifen. *BMC Genomics* 2008; 9: 239.

11 Bos PD, Zhang XH, Nadal C, Shu W, Gomis RR, Nguyen DX *et al*. Genes that mediate breast cancer metastasis to the brain. *Nature* 2009; 459: 1005-1009.

12 Finak G, Bertos N, Pepin F, Sadekova S, Souleimanova M, Zhao H *et al*. Stromal gene expression predicts clinical outcome in breast cancer. *Nat Med* 2008; 14: 518-527.

13 Desmedt C, Piette F, Loi S, Wang Y, Lallemand F, Haibe-Kains B *et al*. Strong time dependence of the 76-gene prognostic signature for node-negative breast cancer patients in

the TRANSBIG multicenter independent validation series. *Clin Cancer Res* 2007; 13: 3207-3214.

14 Curtis C, Shah SP, Chin SF, Turashvili G, Rueda OM, Dunning MJ *et al*. The genomic and transcriptomic architecture of 2,000 breast tumours reveals novel subgroups. *Nature* 2012; 486: 346-352.
