# Supplementary material for: LIMK2 promotes the metastatic progression of triple-negative breast cancer by activating SRPK1
Source: Oncogenesis. 2020 Aug 28;9(8):77. doi: 10.1038/s41389-020-00263-1 (PMC7455732; doi:10.1038/s41389-020-00263-1)
Supplement: Supplementary file 9 — Table S8 [file 41389_2020_263_MOESM9_ESM.docx]

**Table S8:** Reagents and resources used in this study.

| **REAGENT or RESOURCE** | **SOURCE** | **IDENTIFIER** |
| --- | --- | --- |
| **Antibodies** | | |
| ACTINB | Cell signaling | Cat# 4970 (13E5) |
| LIMK2 | Cell Signaling | Cat# 3845 (8C11) |
| LIMK2 | Sigma-Aldrich | Cat# HPA008183 |
| Cofilin | Cell Signaling | Cat# 5175 (D3F9) |
| pCofilin (pS3) | Cell Signaling | Cat# 3313 (77G2) |
| Vinculin | Millipore | Cat# MAB3574 (7F9) |
| SRPK1 | Santa Cruz | Cat# sc-100443 (EE13) |
| SRPK1 | Sigma-Aldrich | Cat# HPA016431 |
| Anti-Phosphoserine Antibody | Sigma-Aldrich | Cat# AB1603 |
| Anti-Phosphoepitope SR proteins antibody, clone 1H4 | Sigma-Aldrich | Cat# MABE50 |
| SRSF3 | Invitrogen | Cat# 33-4200 |
| Alexa Fluor™ 594 Phalloidin | ThermoFisher | Cat# A12381 |
| **Biological Samples** | | |
| Tissue microarray slide (Breast cancer and matched normal breast tissue) | US Biomax, Inc. | Cat# BC081120e |
| Tissue microarray slide (Breast cancer) | Yale Tissue Microarray Facility | Cat# YTMA-311 |
| Tissue microarray slide (Breast cancer) | Yale Tissue Microarray Facility | Cat# YTMA-341 |
| Tissue microarray slide (Breast cancer) | Yale Tissue Microarray Facility | Cat# YTMA-347 |
| **Chemicals, Peptides, and Recombinant Proteins** | | |
| DMEM | GIBCO | Cat# 11965-092 |
| RPMI | GIBCO | Cat# 11875-093 |
| Fetal Bovine Serum | GIBCO | Cat# 10437-028 |
| Trypsin-EDTA | GIBCO | Cat# 25200-056 |
| Penicillin-Streptomycin | GIBCO | Cat# 15140-122 |
| Effectene Transfection Reagent | QIAGEN | Cat# 301427 |
| LX7101 | Cayman | Cat# 21209 |
| TH-257 | Sigma-Aldrich | Cat# SML2275 |
| SRPIN340 | Selleckchem | Cat# S7270 |
| Collagen I, Rat tail | ThermoFisher | Cat# A1048301 |
| Matrigel Basement Membrane Matrix | Corning | Cat# 356237 |
| XenoLight D-Luciferin - K+ Salt Bioluminescent Substrate | Perkin Elmer | Cat# 122799 |
| SRPK1 Recombinant Human Protein | Invitrogen | Cat# PV4215 |
| LIMK2 Recombinant Human Protein | Invitrogen | Cat# PR7519B |
| Cofilin Recombinant Human Protein | Abcam | Cat# ab62958 |
| **Deposited Data** | | |
| Complete list of identified phosphorylated proteins | PRIDE | PXD008246 |
| **Experimental Models: Cell Lines** | | |
| 293T | ATCC | ATCC CRL-3216 |
| MDA-MB-231 | ATCC | ATCC HTB-26 |
| BT-549 | ATCC | ATCC HTB-122 |
| MDA-MB-468 | ATCC | ATCC HTB-132 |
| **Experimental Models: Organisms/Strains** | | |
| Mouse: NSG | Jackson Laboratory | Stock No. 005557 |
| **Oligonucleotides** | | |
| ACTINB qPCR Forward primer | gtcttcccctccatcgtggg |  |
| ACTINB qPCR Reverse primer | cctctcttgctctgggcctc |  |
| LIMK2 qPCR Forward primer | taatccatggggaggtcctg |  |
| LIMK2 qPCR Reverse primer | ctgcgcatcactttcacctc |  |
| SRPK1 qPCR Forward primer | gctggctgcagaagcaacag |  |
| SRPK1 qPCR Reverse primer | agtactgactgcagatccggaag |  |
| **shRNAs** | | |
| LIMK2 | TRCN0000010240 | Cat# RHS3979-9631386 |
| LIMK2 | TRCN0000010242 | Cat# RHS3979-9631388 |
| SRPK1 | TRCN0000001228 | Cat# RHS3979-9569682 |
| SRPK1 | TRCN0000001232 | Cat# RHS3979-9569686 |
| **Recombinant DNA** | | |
| Plasmid: piggyBac GFP-Luc | Ding et al., 2005 | N/A |
| Plasmid: Act-PBase | Ding et al., 2005 | N/A |
| **Commercial assays and kits** | | |
| QCM™ Gelatin Invadopodia Assay | Millipore | ECM671 |
| **Software and Algorithms** | | |
| Prism 8.0 | GraphPad | www.graphpad.com/scientific software/prism |
| ImageJ |  | https://imagej.nih.gov/ij |
| Scaffold | Proteome Software | http://www.proteomesoftware.com/products/scaffold/ |
| R/Bioconductor package dagLogo (v.1.9.2) | Bioconductor | https://www.bioconductor.org/news/bioc_3_9_release/ |
| Ingenuity pathway analysis (IPA) | Qiagen | https://digitalinsights.qiagen.com/products-overview/analysis-and-visualization/qiagen-ipa/ |
